# Synchronous Web-Based Psychotherapy for Mental Disorders From a Health Quality Perspective: Scoping Review

**DOI:** 10.2196/40710

**Published:** 2023-11-03

**Authors:** Raman Dhaliwal, Sidney Yap, Fernanda Talarico, Huda Al-Shamali, Robert Mcweeny, Matthew Reeson, Reham Shalaby, Teresa Chen, Elena Spronk, Rayven Snodgrass, Eileen Tu, Taylor Erick, Tyler Marshall, Megan Kennedy, Andrew J Greenshaw, Olga Winkler, Lisa Burback

**Affiliations:** 1 Department of Psychiatry Faculty of Medicine & Dentistry University of Alberta Edmonton, AB Canada; 2 Faculty of Medicine and Dentistry University of Alberta Edmonton, AB Canada; 3 Geoffrey and Robyn Sperber Health Sciences Library University of Alberta Edmonton, AB Canada

**Keywords:** acceptability, accessibility, app, application, clinical, cognitive, computerized therapy, culture, database, diagnosis, Diagnostic and Statistical Manual of Mental Disorders, DSM, effectiveness, health quality, ICD, International Statistical Classification of Diseases, literature review, mental disorder, mental health, mental, Preferred Reporting Items for Systematic Reviews and Meta-Analysis, PRISMA, privacy, psychoeducation, psychotherapeutic, psychotherapy, psychotherapy, remote delivery, remote psychotherapy, remote, scoping review, security, synchronous, therapist assisted, therapist delivered, therapist.

## Abstract

**Background:**

The COVID-19 pandemic necessitated rapid changes to health care delivery, including a shift from in-person to digitally delivered psychotherapy. While these changes helped ensure timely psychotherapy provision, many concerns exist, including clinical, cultural, practical, privacy, and security issues.

**Objective:**

This scoping review systematically mapped existing peer-reviewed research on synchronous, therapist-delivered web-based psychotherapy for individuals with a diagnosed mental illness. Data were analyzed through the lens of the Alberta Quality Matrix for Health (AQMH) to assess to what degree this literature addresses key indicators of health care quality. This analysis aided in the identification and organization of knowledge gaps with regard to web-based psychotherapies, highlighting potential disparities between previously prioritized dimensions of care and those requiring further attention.

**Methods:**

This review adhered to the PRISMA-ScR (Preferred Reporting Items for Systematic Reviews and Meta-Analyses Extension for Scoping Reviews) guidelines. We included peer-reviewed primary research studies in the English language investigating synchronous, therapist-delivered remote psychotherapy delivered to adults (aged 18 years and older) with a Diagnostic and Statistical Manual of Mental Disorders or International Statistical Classification of Diseases diagnosed mental illness. All other citations were excluded. Relevant studies were identified through MEDLINE, APA PsycINFO, Embase (OVID), Web of Science: Core Collection (Clarivate), Cochrane Library (Wiley), and Scopus (Elsevier) databases. Databases were searched on March 18, 2021. For every publication that was taken into consideration, the data were charted independently by 2 reviewers, and in the event of a discrepancy, the principal investigator validated the choice of either extractor. Results were thematically described according to the 6 AQMH dimensions: acceptability, accessibility, appropriateness, effectiveness, efficiency, and safety.

**Results:**

From 13,209 publications, 48 articles were included, largely from North American studies. Most studies measured treatment effectiveness (n=48, 100%) and acceptability (n=29, 60%) health quality dimensions. Over 80% (40/48) of studies investigated either a cognitive or exposure intervention for either posttraumatic stress disorder or a mood or anxiety disorder, generally indicating comparable results to in-person therapy. Safety (n=5, 10%) was measured in fewer studies, while treatment accessibility, appropriateness, and efficiency were not explicitly measured in any study, although these dimensions were mentioned as a future direction, hypothesis, or potential outcome.

**Conclusions:**

In relation to web-based therapist-delivered psychotherapies for those with a diagnosed mental illness, important aspects of health care quality (accessibility, appropriateness, efficiency, and safety) have received little scientific examination, underscoring a need to address these gaps. There are also significant issues related to the generalizability of this literature, including the underrepresentation of many geographic regions, cultures, populations, clinical contexts, and psychotherapy modalities. Qualitative research in underrepresented populations and settings may uncover important patient and contextual factors important for the future implementation of quality web-based psychotherapy.

## Introduction

### Overview

The COVID-19 pandemic continues to impact the mental health of individuals and communities worldwide [1]. Government-mandated physical distancing restrictions necessitated a rapid shift away from in-person mental health services to digitally delivered services (eg, teletherapy, videoconferencing, eHealth, and mobile health) [2]. Disruptions in routines and activities stemming from the consequences of the pandemic led to increased distress, anxiety, depression, and suicidal ideation both within the general population [3,4] and clinical populations with existing psychiatric disorders [5]. Ensuring the uninterrupted, safe delivery of quality psychotherapy to treat mental disorders, especially in the face of pandemic-related changes, was and continues to be imperative.

Synchronous web-based delivery of mental health services (mental health care delivered by a health care provider over the phone or through web-based videoconference in real time) is not a novel concept [6]. However, caveats surrounding web-based delivery, including clinical, cultural, practical, and privacy and security issues, remain underaddressed [7]. Before the COVID-19 pandemic, several concerns about web-based services slowed widespread implementation, including questions about relative efficacy, the establishment of therapeutic alliances, acceptance of web-based health, technical connectivity challenges, patient privacy and confidentiality, software and equipment availability, usability and reliability, associated costs, and regulatory concerns [8]. While these concerns still exist, the nature of the COVID-19 pandemic created an environment where the implementation of synchronous web-based psychotherapy was accelerated to mitigate potential harms from a lack of in-person services or transmission of the illness.

A recent review reported that psychotherapies delivered through videoconferencing were effective and easy to access for patients; in particular, there was strong evidence supporting the use of cognitive behavioral therapy (CBT) delivered through videoconferencing for posttraumatic stress disorder (PTSD) and depression treatment [9]. That report did not fully address other concerns about web-based psychotherapies (such as patient safety, acceptance of web-based psychotherapy by patients and clinicians, and training required to use web-based platforms). These research gaps may contribute to mental health clinicians favoring in-person services more often than web-based services [10], exacerbating clinician hesitancy to provide web-based care. A pre–COVID-19 study investigating why psychologists did not use telepsychology in their practice found that concerns surrounding insufficient training, patient safety, and privacy issues were the main deterrents [11].

Research interest in this area has grown following the COVID-19 pandemic, with new evidence that synchronous web-based psychotherapy can be implemented successfully in a variety of contexts. Acceptance and use of this modality, therefore, are likely to continue to increase over time, necessitating closer analysis of the quality of web-based services and ongoing knowledge and research gaps. As such, we chose to analyze the literature through the lens of the Alberta Quality Matrix for Health (AQMH) [12], a standardized and validated tool providing holistic evaluation of health systems from patient, provider, and institutional perspectives. See Multimedia Appendix 1 for a copy of the AQMH.

### Rationale

The AQMH was created following extensive consultation by the Health Quality Council of Alberta, Canada, to provide a framework for organizing information and thinking regarding the complexity of health systems [13]. The AQMH has the following two components: (1) dimensions of quality, which focuses on aspects of the patient and client experience; and (2) areas of need, which divides services provided by the health system into 4 distinct but related categories (being healthy, getting better, living with illness or disability, and end of life).

In this scoping review, we assess whether peer-reviewed studies addressed the following six dimensions of the AQMH with respect to synchronous, clinician-delivered web-based psychotherapy for mental disorders:

1. Acceptability: health services are respectful and responsive to user needs, preferences, and expectations.
2. Accessibility: health services are obtained in the most suitable setting at a reasonable time and distance (for this paper, accessibility is analogous to access to care and does not refer to designing services for people with disabilities).
3. Appropriateness: health services are relevant to user needs and are based on accepted or evidence-based practice.
4. Effectiveness: health services are based on scientific knowledge to achieve desired outcomes.
5. Efficiency: resources are optimally used in achieving desired outcomes.
6. Safety: services mitigate risks to avoid unintended or harmful results.

We used the AQMH as a frame for this literature review to help identify and organize knowledge gaps with regard to web-based psychotherapies, highlighting potential disparities between previously prioritized dimensions of care and those requiring further attention.

### Objectives

This scoping review aims to systematically map, through the lens of the AQMH, the existing peer-reviewed research on synchronous, therapist-delivered web-based psychotherapy for individuals with a mental illness, diagnosed according to the Diagnostic and Statistical Manual of Mental Disorders (DSM) or International Statistical Classification of Diseases (ICD). In doing so, the review strived to identify the scope of the literature related to each dimension of health quality, methodologies used, and research gaps.

## Methods

### Protocol

This review followed the PRISMA-ScR (Preferred Reporting Items for Systematic Reviews and Meta-Analysis Extension for Scoping Reviews) [14]. The protocol was not registered a priori.

### Eligibility Criteria

#### Overview

This scoping review included peer-reviewed primary studies investigating synchronous (video, voice, or voice and avatar) therapist-delivered remote psychotherapy delivered to adults (aged 18 years and older) with a DSM- or ICD-diagnosed mental illness. Studies in the English language with quantitative (randomized controlled trials [RCTs], quasi-experimental, and observational studies) and qualitative methodologies were considered for inclusion. Synchronous, therapist-delivered remote psychotherapy refers to psychotherapy that is delivered in real time by a therapist who is not in the physical presence of the participant.

Studies were excluded if they focused on application-based and fully computerized interventions (eg, internet-based CBT); chat groups; support groups; self-directed psychotherapy; therapist-facilitated, guided, or supported interventions; blended interventions (eg, therapist-guided internet-based CBT); engagement-only interventions (eg, psychoeducation, motivational interviewing, rapport building, strategic brief intervention, and referral to treatment); and consultation- or assessment-only interventions. Studies not conducted in the English language were excluded.

#### Justification for Eligibility Criteria

The reason for this review’s focus on fully human-delivered interventions is that evidence suggests that therapeutic alliance is an important predictor of psychotherapy success [15]. Furthermore, this review focused on therapist-delivered web-based psychotherapy because (1) therapists deliver most psychotherapy in health care systems; (2) during the COVID-19 pandemic, web-based care quickly became the norm despite apprehension among therapists; and (3) questions remain about the relative quality of in-person versus web-based interventions [16].

Because we sought to focus on the specific issue of synchronous, therapist-delivered remote psychotherapies, multiple interventions were excluded, including chat groups, support groups, self-directed psychotherapy, text-based interventions, and fully computerized psychotherapeutic interventions. App-based interventions have also already been extensively reviewed in the literature [17]. Therapist-assisted interventions, where the therapist is guiding or supporting an intervention but not fully delivering psychotherapy, and blended interventions (where there is a mix of apps, computerized therapy, and therapist intervention) were excluded, as the purpose of this review was to specifically examine the impact of remote delivery rather than novel treatments. Engagement-, consultation-, or assessment-only interventions were excluded because this review focused specifically on the impact of psychotherapeutic treatments. Case reports were excluded because of their limited generalizability. Articles in a language other than English were excluded for feasibility reasons.

### Information Sources

An expert health research librarian searched 7 electronic health databases for relevant studies from database inception until March 18, 2021. Databases searched comprised: MEDLINE, APA PsycINFO, Embase (OVID), Web of Science: Core Collection (Clarivate), Cochrane Library (Wiley), and Scopus (Elsevier). The search process was documented and reported in adherence to PRISMA-S (Preferred Reporting Items for Systematic Reviews and Meta-Analysis for Searching) guidelines.

### Search Strategy

The search process was documented and reported in adherence to the PRISMA-S extension [18]. Methodological guidance for the systematic search was sought from the JBI Manual for Evidence Synthesis, Chapter 11: Scoping Reviews [19].

The search strategy was derived from three main concepts: (1) psychotherapy; (2) mental health diagnoses, as defined by the DSM or ICD; and (3) web-based or remote delivery or telemedicine. Search terms were developed in consultation with an experienced health sciences librarian at the John W Scott Health Sciences Library at the University of Alberta, using a combination of database medical subject headings and relevant keywords. To enhance search sensitivity, no limits, such as publication date, were applied to the searches. The search strategies and key terms for all databases can be found in Multimedia Appendix 2.

### Study Selection

**Figure 1:**
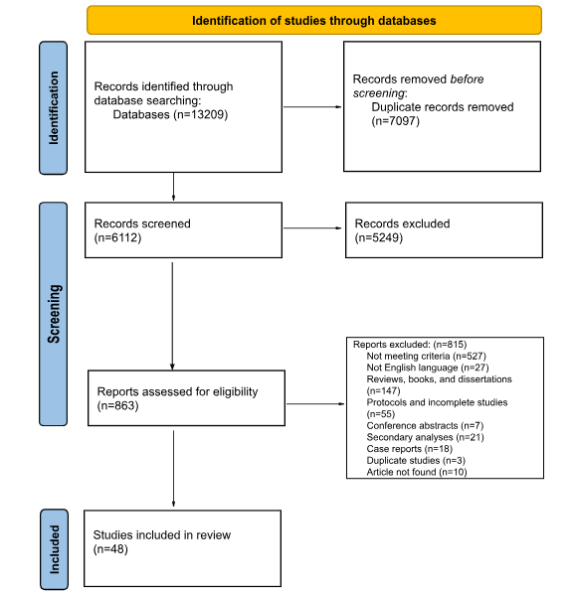
PRISMA (Preferred Reporting Items for Systematic Reviews and Meta-Analysis) flow diagram.

Results of the database searches were uploaded into Covidence software (Veritas Health Innovation) [20], which removed duplicate citations. Before title and abstract screening of citations, team members reviewed 100 citations to ensure consistency among reviewers. Then, 2 reviewers, each independently, screened the titles and abstracts of identified citations, with conflicts resolved by a third independent reviewer. After excluding citations in the title and abstract screening phase, articles were selected for full-text review. The same process was followed, with pilot-testing followed by 2 independent reviewers screening each citation and a third independent reviewer resolving conflicts, with team discussions occurring as needed. Articles were then selected for inclusion in the scoping review. The reference lists of these relevant articles, as well as review articles already familiar to the authors, were hand-searched to look for additional relevant publications. See Figure 1 for more details.

### Data Extraction and Analysis

Following abstract screening and full-text review stages, team members pilot-tested a Google Form for data extraction. The pilot involved all reviewers extracting data from a random sample of included articles. The form was revised as necessary under the supervision of the principal investigator (PI) before formal data extraction began. With assistance from the PI, reviewers then constructed and revised a suitable data extraction table. The 2 lead reviewers independently charted the data from each publication. In the case of a discrepancy between 2 data extractions, a PI validated the data using the source material.

The following information was extracted from the included literature: author country, study design, inclusion and exclusion criteria, sample size, mean age, sex and gender, race or ethnicity, DSM or ICD diagnosis, data analysis strategies, outcome measures, dropouts, results, reported barriers and facilitators to the use of remote psychotherapy, recommendations, and study limitations. Specific information about the intervention (eg, description of the intervention, mode of delivery [group or individual therapy delivery], and training or background of clinicians) was also extracted. Additionally, data were extracted regarding whether the study addressed each AQMH dimension, including treatment acceptability, accessibility, appropriateness, effectiveness, efficiency, and safety.

As part of the data analysis strategy, the study team, under the guidance of the PI and co-PI, evaluated the relevance of the included studies’ outcomes, aims, and hypotheses in the context of AQMH dimensions. Following this, a thematic qualitative analysis was conducted to elucidate notable themes [21]. Articles that assessed the same AQMH dimension were collated and analyzed together, as presented in the results section. The concept of therapeutic alliance, while related to effectiveness, was analyzed under the quality dimension of acceptability because, within the AQMH framework, acceptability “includes qualities such as compassion, empathy, and responsiveness and refers to care and service that establishes a partnership between providers, patients, clients, and their families (when appropriate) to ensure decisions respect patients’ or clients’ wants, needs, and preferences.” Therefore, acceptability in the AQMH includes the relational and collaborative aspects of care inherent in the concept of therapeutic alliance [13,22].

## Results

### Search Results

The search strategy yielded 13,209 publications, of which 7097 duplicate articles were removed. The titles and abstracts of 6112 articles were reviewed. After excluding 5249 citations, 863 articles were selected for full-text review, with 815 articles being excluded. The remaining 48 articles were included in this review [23-70]. See Table S1 in Multimedia Appendix 3 and Figure 1 for details.

### Study and Population Characteristics

The articles included in this review enrolled a total of 4872 participants. The majority (n=46, 96%) of studies collected only quantitative data, with 2 (4%) mixed methods studies collecting both quantitative and qualitative data. Study designs included RCTs (n=32, 67%), non-RCTs (n=8, 17%), pretest-posttest designs (n=6, 13%; no control group), and other study designs (n=2, 4%). Most (n=37, 77%) studies were published in the past 10 years (from 2011 to 2020), and 83% (n=40) were published in the United States or Canada. The predominant DSM and ICD categories were PTSD (n=19, 40%), depressive disorders (n=11, 23%), and anxiety disorders (n=10, 21%). The vast majority (n=14, 74%) of the PTSD studies were conducted in military populations. All studies reported participants as being either male or female, with a total of 52.3% (2364/4520) male and 47.7% (2156/4520) female participants enrolled across studies, and none reported on gender-diverse populations. A total of 33 (69%) studies reported on participants’ race or ethnicity, with 58.2% (n=1937) of these studies’ participants identifying as Caucasian. All studies were conducted within an outpatient setting, with two-thirds enrolling civilians and one-third involving military populations (Tables S1-S5 in Multimedia Appendix 2).

### Intervention Characteristics

Most psychotherapies were delivered in a one-on-one setting (n=44, 92%), with few studies using group therapy (n=4, 8%). Psychotherapy was predominantly delivered through videoconferencing (n=31, 65%; including, but not limited to Tandberg videoconference system: n=10; Skype: n=3; Cisco Webex: n=2; Polycom: n=2; KMEA TV500SP: n=1; iChat through Mac computers: n=1; Facetime through iPad 2: n=1), or telephone (n=15, 31%). The most common psychotherapy interventions included CBT (n=27, 56%), exposure therapy or prolonged exposure (n=7, 15%), and cognitive processing therapy (n=6, 13%; Table S6 in Multimedia Appendix 2).

### AQMH Dimensions

Most included studies measured treatment effectiveness (n=48, 100%) and acceptability (n=29, 60%). Treatment safety (n=5, 10%) was measured in a smaller number of studies. Treatment accessibility (n=44, 92%), appropriateness (n=2, 4%), and efficiency (n=2, 4%) were mentioned in certain studies (ie, discussed as a future direction, hypothesis, or potential outcome), but none of these latter dimensions were measured in any of the included studies (Table 1).

Studies measuring effectiveness did so using a variety of outcome measures (eg, self-report or clinician-rated questionnaires assessing symptom severity or changes in the frequency of psychiatric episodes following treatment). Common outcome measures included the Beck Depression Inventory (n=18, 38%), the PTSD checklist (n=10, 21%), the Clinician-Administered PTSD Scale (n=9, 19%), the Patient Health Questionnaire-9 (n=6, 13%), the Generalized Anxiety Disorder Scale 7-items (n=4, 8%), the Penn State Worry Questionnaire (n=4, 8%), the State-Trait Anxiety Inventory (n=4, 8%), and the Hamilton Depression Rating Scale (n=4, 8%; Table S2 in Multimedia Appendix 3). A total of 9 (19%) studies used only self-report validated scales, while 1 (2%) study used only clinician-administered validated scales. Both self-report and clinician-administered outcome measures were used in 38 (79%) studies. Most studies reported that remote delivery of psychotherapy was not inferior to in-person delivery of psychotherapy (n=32, 67%), whereas 12 (25%) studies did not have an in-person comparator but reported positive results. Overall, 4 (8%) studies found that remote delivery of psychotherapy was inferior to in-person delivery (Table S7 in Multimedia Appendix 2).

**Table 1:** Alberta Quality Matrix for Health (AQMH) dimensions.

AQMH dimension of quality	Studies measuring the AQMH dimension, n (%)	Studies mentioning the AQMH dimension as a potential outcome, n (%)	Studies mentioning the AQMH dimension as a future direction or hypothesis, n (%)
Acceptability	29 (60) [23-27,29,31,35-40,42-44,50,51,54-57,59,61,65,66,68-70]	3 (6) [33,46,63]	0 (0)
Accessibility	0 (0)	14 (29) [30,31,36,38,39,44,46,53-56,64,68,70]	30 (63) [23-29,32-35,37,40-42,47-51,57-59,61-63,65-67,69]
Appropriateness	0 (0)	2 (4) [44,46]	0 (0)
Effectiveness	48 (100) [23-70]	0 (0)	0 (0)
Efficiency	0 (0)	2 (4) [48,65]	0 (0)
Safety	5 (10) [43,53,58,59,63]	5 (10) [42,54-57]	1 (2) [62]

Studies measuring treatment acceptability (n=29) also used a variety of instruments to assess patient-therapist relationship or patient satisfaction with treatment. The full or short-form version of the Working Alliance Inventory was used in 55% (n=16) of studies measuring treatment acceptability. Other validated scales used to assess treatment acceptability included the Client Satisfaction Questionnaire (n=5, 18%), the Charleston Psychiatric Satisfaction Scale (n=4, 14%), the Telemedicine Satisfaction and Acceptance Scale (n=4, 14%), the Group Therapy Alliance Scale (n=3, 11%), the Distance Communication Comfort Scale (n=2, 7%), the Videoconference Therapy Questionnaire (n=2, 7%), the Service Delivery Perceptions Questionnaire (n=2, 7%), the California Psychotherapy Alliance Scale (n=1, 4%), the Client Satisfaction Questionnaire (n=1, 4%), the Reaction to Treatment Questionnaire (n=1, 4%), and the Patient Satisfaction Survey (n=1, 4%; Table S2 in Multimedia Appendix 3). Nonvalidated surveys were used to assess acceptability in 25% (n=7) of studies. Additionally, 3 (6%) studies mentioned acceptability as a potential outcome without actually measuring acceptability. These studies indicated that age, experience, and positive attitudes toward videoconferencing may be factors modifying treatment acceptability [33,46], and that veteran populations may be more amenable to the web-based delivery format [63].

Studies that measured safety did so by tracking the number of adverse events or safety issues that arose during treatment, including suicidal ideation and suicide attempts, rather than validated scales. All studies that measured safety (n=5, 10%) reported no adverse events or safety issues related to web-based psychotherapy. Safety was also mentioned as a potential outcome in 5 (10%) studies and as a future direction in 1 (2%) study.

Treatment accessibility was mentioned as a potential outcome in 29% (n=14) of studies and as a future direction or hypothesis in 63% (n=30) of studies. These studies discussed access to psychotherapy in various populations, including rural veterans [54-56], psychiatric outpatients living in remote areas [30], and ethnically diverse women living in underserved rural and urban areas [31]. Treatment appropriateness and efficiency were mentioned as potential outcomes of interest in 4% (n=2) of studies. However, none of the studies measured these dimensions.

## Discussion

### Principal Results

Of 13,209 publications, 48 studies were included for final review. Of the 6 AQMH dimensions, most studies measured treatment effectiveness (n=48, 100%) and acceptability (n=29, 60%). Treatment safety was infrequently (n=5, 10%) reported on, while no studies measured accessibility, appropriateness, and efficiency, despite many papers mentioning their importance as a future direction, hypothesis, or potential outcome. This review, therefore, uncovers significant knowledge gaps regarding the quality of remotely delivered synchronous psychotherapy for those with mental illnesses.

### Effectiveness

The AQMH dimension of effectiveness was investigated in all studies meeting review criteria, most of which were published in the past 10 years. This is not surprising, given its fundamental importance to treatment, the rapidly growing and relatively nascent nature of this practice, the brisk evolution of technologies, and the impact of the COVID-19 pandemic [71]. Most included studies reported significant improvements in symptoms of depression, PTSD, and anxiety and the noninferiority of therapist-delivered web-based psychotherapy compared to in-person delivery. However, it is important to note that 83% (40/48) of studies included either a cognitive or exposure intervention, which is not representative of psychotherapy in general.

A wide variety of common, empirically supported psychotherapies are represented in this review, such as dialectical behavior therapy and eye movement desensitization and reprocessing. It is conceivable that important nuances are lost if these results are generalized across all modalities. For example, eye movement desensitization and reprocessing requires the application of dual attention tasks, such as eye movements, that might be impacted by screen size or internet quality. Similarly, dialectical behavior therapy is often used for those with borderline personality disorder, who may have greater interpersonal sensitivity and therefore a different response to remotely delivered psychotherapy [72]. Alternative therapeutic contexts, such as group therapy, were also underrepresented despite validated in-person efficacy [73]. Therefore, while data for cognitive and exposure therapies are promising, more research may be required to understand the effectiveness of remote delivery of other psychotherapy modalities.

The same generalizability concerns apply to the populations studied. Over 80% (40/48) of studies focused on patients with PTSD and depressive or anxiety disorders, which does not reflect the spectrum of mental health challenges encountered in psychiatric practice. Effectiveness may be impacted by characteristics of other mental illnesses not represented in this review. These populations may also have unique mental health and sociocultural needs impacting their dispositions toward, or away from, web-based psychotherapy. For example, web-based psychotherapy might reach those hesitant to leave their home, such as those with social anxiety or agoraphobia; however, this could also conceivably limit effectiveness if the exposure involved in traveling to a clinic is part of the benefit of therapy. Web-based therapy might be useful for those with serious mental illnesses, improving access and allowing information about their home context while limiting subtle monitoring of mental status by the therapist. Serious mental illness is also associated with a greater prevalence of cognitive deficits, which may interact with the web-based context, especially if there are distractions in the home environment.

### Acceptability

Most included studies reported that web-based psychotherapy provided similar treatment acceptability to in-person psychotherapy based on quantitative measures (Multimedia Appendix 3). Many of these studies reported that participants had comparable opinions of therapeutic alliance between web-based and in-person psychotherapy, suggesting that therapeutic alliance was not necessarily impacted by web-based delivery, at least for the populations studied. This is particularly encouraging, as the therapeutic relationship has been found to correlate highly with client outcome, where a strong relationship would likely lead to positive therapeutic results [74]. Participant selection bias may have contributed to this finding if participants were already accepting of web-based mental health services before their respective interventions. For example, one-third of the included studies were conducted in military populations, who may be more open toward web-based psychotherapy due to fear of encountering trauma-related cues at in-person clinics [64]. Studies in other populations are needed for the generalizability of these findings.

Most studies addressed the acceptability of web-based psychotherapy from the client’s perspective and did not examine the acceptability of web-based psychotherapy from the therapist’s perspective. This gap in research should be addressed, as much of the hesitancy regarding wider use and implementation comes from therapists themselves [74].

### Appropriateness

Treatment appropriateness, defined as whether services are relevant to users’ needs and are based on accepted or evidence-based practice, was mentioned as a potential outcome by only 2 studies. While not stated explicitly in the AQMH, treatment appropriateness has implications for equity, diversity, and inclusion within the health care system. For example, one of the reviewed studies that mentions treatment appropriateness emphasized that “culturally appropriate examples and language were used to reflect life experiences and context among African American women” [45]. However, most reviewed studies did not account for gender and cultural identity, economic background, or other equity, diversity, and inclusion factors to improve treatment appropriateness for participants, which may impact other indices of quality (acceptability, effectiveness, accessibility, and safety) [75-77].

Web-based delivery of psychotherapies presents opportunities to deliver care that is appropriately tailored to population needs and preferences. For example, in rural or isolated communities, web-based psychotherapy may effectively link patients with appropriate mental health clinicians who are responsive to their personal sociodemographic context and mental health needs. This overlaps with accessibility in that some underserved populations may live in more remote areas with less physical access to appropriate psychotherapeutic care. Additionally, web-based psychotherapies may have the potential to be conducted within the patient’s preferred therapeutic environment (one-on-one therapy vs group therapy) more readily than in-person therapy. Further, previous research has found that differences in cultural interpretations of certain social behaviors may affect treatment appropriateness [75]. For example, White and African American populations in the United States generally interpret direct eye contact as a sign of respect, while individuals of Native American, Hispanic, and Asian cultures may consider direct eye contact to be disrespectful or rude [78]. The use of web-based platforms may ease the challenges of matching clients with culturally sensitive and appropriate therapists. Finally, some individuals may prefer videoconferencing-based interventions over telephone-based interventions, or vice versa, underscoring the flexibility of synchronous web-based modalities for appropriately tailored treatments. This flexibility creates opportunities for further investigation into appropriate use cases and outcomes for all applicable populations, including patients and administering clinicians.

### Accessibility

While treatment accessibility was mentioned as a potential outcome, future direction, or hypothesis in many of the reviewed studies, no studies measured this dimension. This indicates that treatment accessibility was not a primary focus of the reviewed studies, and research is needed to explore this dimension of web-based psychotherapy. The lack of literature evaluating accessibility may be a result of the absence of validated research instruments to measure quantitative differences in treatment accessibility between modes of delivery. Regardless, future research should address this gap. Data regarding differences in timeliness, financial expense, and support for accessing in-person or web-based psychotherapy may be useful in addressing issues of accessibility. Doing so may allow researchers and policy makers to better understand the unique advantages of web-based delivery for certain patients and practitioners.

It is possible that certain populations may benefit more than others from increased access to web-based psychotherapy (eg, individuals with child-care responsibilities, individuals with mobility issues or barriers to reliable transportation, and residents of rural communities). However, not all individuals have equal access to adequate resources that support or enable web-based psychotherapy, such as stable high-speed internet, a computer or telephone, technological support, or a private and quiet enough space to derive benefit from web-based psychotherapy. Such inequities may stem from differences in socioeconomic status, geocultural factors, age, and technological literacy. These factors should be considered in future research to ensure that increasing web-based psychotherapy prevalence does not create further disparities in treatment accessibility.

### Efficiency

Treatment efficiency, the optimal use of resources to achieve outcomes, was only mentioned in 2 included studies. This gap may stem from the complexities involved in making comparisons about how resources are used within different therapeutic environments and who shoulders the burdens. For example, the manner and degree to which web-based delivery imposes or alleviates financial, time, and other burdens for therapists, patients, and systems depend on many contextual factors, such as location, training, regulatory, technological, and policy factors, and whether care is delivered within a private or publicly funded setting. For example, therapists delivering web-based psychotherapies at mental health clinics may have different access to personnel, technologies, and financial resources compared to therapists delivering care from home or in private practice. These considerations are likely to change over time as available technologies, training, and costs change. Direct comparisons of these factors between in-person and remotely delivered therapy were not apparent in the studies included in this review, and we encourage novel targeted research into this topic. In addition, more research focus should be granted to group psychotherapies in this context; such interventions may be more resource-efficient, as 1 or 2 therapists could provide care to multiple patients at once.

Many opportunities exist to better understand the impact of web-based psychotherapies on treatment efficiency. Cost impact analyses could allow researchers and policy makers to formally quantify the impacts of web-based psychotherapy according to efficiency metrics, including money, time, and treatment completion. Further, research on efficiency and other AQMH dimensions across private and public settings could inform quality indicators or benchmarks relevant to public funding of psychotherapy services, including within primary care and private practices.

### Safety

Treatment safety was another underexplored AQMH dimension, representing a critical gap for treating psychiatric disorders, especially in the context of suicidality. There was no consistency among the included studies regarding how safety was defined and evaluated; for example, some studies recorded suicide attempts and suicidal ideation over the course of the intervention as a measure of treatment safety, while others did not operationalize or record safety. Important research opportunities include studying contextual factors, such as patient or intervention characteristics, and establishing standardized operational definitions of safety, respective of mental health contexts, to better support objective comparisons of safety between web-based and in-person psychotherapies.

Overall, 5 of the included studies measured safety, but no safety issues were reported. This may originate from possible selection bias. We found that 11 studies excluded individuals with suicidal ideation, and none included populations with personality disorders. These populations are often associated with greater safety risks, service needs, and challenges in forming and maintaining a therapeutic alliance [79]. It is unknown if web-based psychotherapy elevates safety risks for certain high-risk patient populations. Similarly, the barriers and facilitators for clinician confidence and capacity to assess and manage safety digitally, given the lack of physical presence in the patient’s environment, are understudied [80,81]. To ameliorate hesitancy among clinicians regarding web-based psychotherapy in high-risk populations, it may be necessary to expand protocols and training programs that assist clinicians with risk mitigation in digital contexts. Ensuring that mental health clinicians have a clear understanding of when and how to use web-based psychotherapies for optimal patient safety should be a priority moving forward.

### Future Directions

The previous discussion revealed various important research gaps related to web-based psychotherapy that may mask essential details vital for health care quality. Given the interplay between quality dimensions, patients, therapists, and contextual factors and the difficulty of capturing such nuances with quantitative measures, we advocate for increased qualitative research. Such research would expand upon existing patient satisfaction data, allowing for a more comprehensive understanding. Neglecting the lived experiences of patients and practitioners may deny clinicians and policy makers crucial insights about how to optimize the quality of web-based psychotherapy services.

We also recommend a focus on addressing issues of generalizability, including across psychotherapy modalities, psychiatric diagnoses, and populations. As most studies were primarily conducted in the United States and Canada, future research should be conducted in more diverse geographic locations and across cultures to better capture the needs of different sociodemographic groups and understand factors impacting the viability of web-based psychotherapies in different locations. This research would further clarify the treatment accessibility, appropriateness, and efficiency of web-based psychotherapies within a worldwide context and across categories important for equity, diversity, and inclusion.

Ultimately, the majority of AQMH dimensions are underexplored, hindering policy development and clinical optimization. The need for improved research on health quality exists for both traditional in-person practice and web-based settings. However, the specific challenges of web-based settings remain ill-defined and require prompt attention, given the fast-paced evolution of policies and practices and their potential impact on patients, clinicians, and health care systems.

### Strengths and Limitations

The current review has many strengths. This review adhered to PRISMA-ScR guidelines and searched multiple databases for relevant literature. Rigorous quality controls were used to ensure that consensus was reached during data extraction and synthesis.

We also acknowledge several potential limitations with this review. First, the AQMH may not consider all dimensions of health care quality in end-user contexts. For example, high-quality clinician training and general practice guidelines are needed to create and maintain high-quality services over the long term; such aspects are not explicitly addressed. In addition, the AQMH was not specifically created for psychotherapy and therefore may not be optimal for evaluating certain psychotherapy-related factors, such as therapeutic alliances. Further, the strength of the AQMH is for evaluating health care quality and may not be a good framework for addressing other systemic issues such as the “fit” of interventions within health systems across different contexts (eg, public and private). Further research evaluating the AQMH from different health-context perspectives may be useful to diversify considerations and the applicability of the AQMH.

Second, the included studies lacked diversity in certain areas (see Future Directions section), including the geocultural diversity of the study populations. Thus, our scoping review is limited to the populations reported in the source studies. Our search was limited to papers written in English, which may have limited our ability to review all relevant literature.

Third, our results are limited to certain clinical contexts. This review included research that investigated synchronous, therapist-delivered remote psychotherapy, limiting generalizability to mental health care contexts that use app-based or blended interventions. We therefore cannot make comparisons between therapist-delivered remote psychotherapies and adjacent interventions. By extension, included papers seldom reported on the funding for participants’ web-based psychotherapy sessions (eg, out-of-pocket costs and degree of public or private insurance or billing). Thus, we cannot speak to whether the context (private or public) of remote therapist-delivered psychotherapy impacts these services in the context of the AQMH. This limitation may speak to the difficulties in applying research study findings to specific interventions in complex health care systems.

### Conclusions

Current research investigating web-based psychotherapy delivery for those with mental illness has largely focused on treatment acceptability and effectiveness. This review identified multiple gaps within the existing literature: (1) effectiveness data are largely limited to cognitive-behavioral and exposure-based interventions for PTSD, mood disorders, and anxiety disorders; (2) there is a lack of qualitative research; (3) little research is focused on treatment appropriateness, efficiency, and safety; (4) the geographical range of research is limited; and (5) research has been limited to certain clinical contexts. Without addressing these limitations, we run the risk of generalizing research findings that do not apply to the broader population with mental illnesses. In contrast, bridging these gaps would greatly increase our understanding of the strengths and weaknesses of web-based psychotherapy delivery, including what works for whom and in what context. Without such knowledge, we risk creating new inequities within the health care system that may lead to health care providers providing substandard care in several domains. To ensure that quality care is provided to those receiving web-based psychotherapies, future research should address gaps identified in this review using validated health-quality tools and frameworks, such as the AQMH.

## Appendix

Multimedia Appendix 1 Alberta Quality Matrix for Health.

Multimedia Appendix 2 Search strategy and additional tables.

Multimedia Appendix 3 Characteristics and results of reviewed studies.

Multimedia Appendix 4 PRISMA-ScR checklist.

## Abbreviations

AQMH: Alberta Quality Matrix for Health
CBT: cognitive behavioral therapy
DSM: Diagnostic and Statistical Manual of Mental Disorders
ICD: International Statistical Classification of Diseases
PI: principal investigator
PRISMA-S: Preferred Reporting Items for Systematic Reviews and Meta-Analysis for Searching
PRISMA-ScR: Preferred Reporting Items for Systematic Reviews and Meta-Analysis Extension for Scoping Reviews
PTSD: posttraumatic stress disorder
RCT: randomized controlled trial

## Data Availability

All data generated or analyzed during this study are included in this published article (and its supplementary information files).
