# Supplementary figures and images for: Synchronous Web-Based Psychotherapy for Mental Disorders From a Health Quality Perspective: Scoping Review
Source: J Med Internet Res. 2023 Nov 3;25:e40710. doi: 10.2196/40710 (PMC10656669; doi:10.2196/40710)

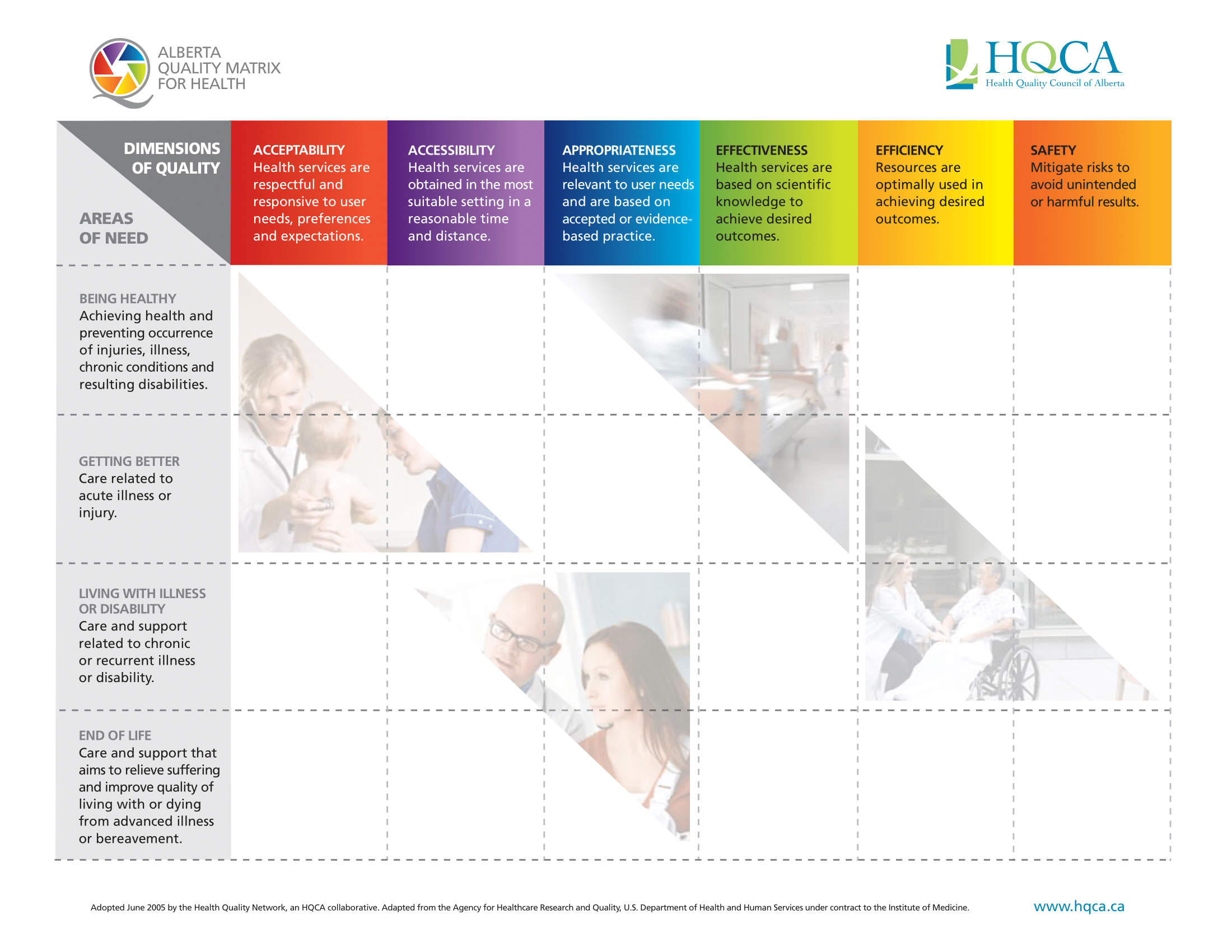

Supplement: Multimedia Appendix 1 [file jmir_v25i1e40710_app1.png]
