# Supplementary material for: Synchronous Web-Based Psychotherapy for Mental Disorders From a Health Quality Perspective: Scoping Review
Source: J Med Internet Res. 2023 Nov 3;25:e40710. doi: 10.2196/40710 (PMC10656669; doi:10.2196/40710)
Supplement: Multimedia Appendix 2 [file jmir_v25i1e40710_app2.docx]

**Multimedia Appendix 2**

**Part 1: Final search strategies for all databases**

**Database: Medline via OVID (1946 - Present)**

**Date of search: July 20 2020**

1 psychotherapy/ 54032

2 exp cognitive behavioral therapy/ 28453

3 (psychotherap* or "psycho-therap*").ti,ab,kf. 46645

4 (cognitive adj2 "behavio?r* therap*").ti,ab,kf. 17565

5 or/1-4 106653

6 exp mental disorders/ or exp anxiety disorders/ or exp obsessive-compulsive disorder/ or exp phobic disorders/ or exp "bipolar and related disorders"/ or exp "disruptive, impulse control, and conduct disorders"/ or exp dissociative disorders/ or exp elimination disorders/ or exp enuresis/ or exp "feeding and eating disorders"/ or exp mood disorders/ or exp depressive disorder/ or exp neurocognitive disorders/ or exp neurodevelopmental disorders/ or exp "attention deficit and disruptive behavior disorders"/ or exp tic disorders/ or exp paraphilic disorders/ or exp personality disorders/ or exp "schizophrenia spectrum and other psychotic disorders"/ or exp psychotic disorders/ or exp schizophrenia/ or exp sexual dysfunctions, psychological/ or exp sleep wake disorders/ or exp dyssomnias/ or exp parasomnias/ or exp somatoform disorders/ or exp substance-related disorders/ or exp alcohol-related disorders/ or exp narcotic-related disorders/ or exp substance withdrawal syndrome/ or exp "trauma and stressor related disorders"/ or exp stress disorders, traumatic/ 1294085

7 suicidal ideation/ 7077

8 ((psychological or psychiatric or mental*) adj2 (disorder* or ill or illness* or health or wellness)).ti,ab,kf. 265669

9 (behavio?r adj2 disorder*).ti,ab,kf. 7668

10 (((anxiety or panic or "obsessive-compulsive" or phobi*) adj1 disorder*) or OCD).ti,ab,kf. 50695

11 ((depressi* or bipolar or mood) adj1 disorder*).ti,ab,kf. 77986

12 ("eating disorder*" or anorexi* or bulimi* or "binge eat* disorder*" or pica or "ruminat* disorder*" or "food intake disorder*" or ARFID).ti,ab,kw. 52015

13 "personality disorder*".ti,ab,kf. 20141

14 ("post traumatic stress disorder*" or PTSD).ti,ab,kf. 29420

15 ("psychotic disorder*" or schizophreni*).ti,ab,kf. 131837

16 ((suicid* or selfharm* or "self harm*") adj2 (idea* or thought*)).ti,ab,kf. 13848

17 or/6-16 1506479

18 Telemedicine/ or (telecare or telecollaborat* or teleconference* or telehealth or teleguide*or telediagnos* or telemed* or telemonitor* or telepresence* or "telemental health" or telepsychotherap* or ((skype or facetime or zoom or internet or web or online or web or video or distan* or remote*) adj2 (deliver* or conferenc* or call*)) or ehealth* or tele care or tele collaborat* or tele conference* or tele health or tele guide* or tele diagnos* or tele med* or tele monitor* or tele presence* or "tele mental health" or "tele psychotherap*" or (teletherap* not (x-ray or radiat* or cobalt or gamma* or cesium))).mp. 39528

19 ((distan* or remote* or video* or virtual* or tele or web or internet or online or technology or "text messag*") adj4 (psychotherap* or "mental health" or CBT or (cognitive adj2 "behavio?r* therap*"))).mp. 2897

20 Telephone/ 11763

21 ((telephone* or telephoning or phone* or phoning) adj2 (call* or deliver* or psychotherap*)).ti,ab,kf. 8279

22 or/18-21 58465

23 5 and 17 and 22 2153

24 (note* or comment* or editorial* or news* or opinion or letter).pt. 2069060

25 23 not 24 2101

26 remove duplicates from 25 2093

**Database: EMBASE via OVID (1974 - Present)**

**Date of search: July 20 2020**

1 psychotherapy/ 85537

2 exp cognitive behavioral therapy/ 13012

3 (psychotherap* or "psycho-therap*").ti,ab,kw. 60610

4 (cognitive adj2 "behavio?r* therap*").ti,ab,kw. 25434

5 or/1-4 130186

6 mental disease/ or exp addiction/ or adjustment disorder/ or alexithymia/ or exp anxiety disorder/ or behavior disorder/ or delirium/ or exp dissociative disorder/ or emotional disorder/ or exp mood disorder/ or exp neurosis/ or exp personality disorder/ or exp psychosexual disorder/ or exp psychosis/ or exp psychosomatic disorder/ or exp psychotrauma/ or exp schizophrenia spectrum disorder/ or thought disorder/ or exp depression/ or exp mania/ or exp bipolar disorder/ or exp perinatal depression/ or exp gender dysphoria/ or exp hypersexuality/ or exp paraphilic disorder/ or exp alcohol psychosis/ or exp delusion/ or exp drug induced psychosis/ or exp hallucination/ or exp paranoid psychosis/ or exp schizophrenia/ or exp somatoform disorder/ or exp body dysmorphic disorder/ or exp conversion disorder/ or behavioral addiction/ or drug dependence/ or withdrawal syndrome/ or drug dependence/ or exp alcoholism/ or exp narcotic dependence/ or exp alcohol withdrawal syndrome/ or exp delirium tremens/ or exp obsessive compulsive disorder/ or exp phobia/ or exp hoarding disorder/ or exp obsession/ or exp attention deficit disorder/ or automutilation/ or exp disruptive behavior/ or exp eating disorder/ or pica/ or exp impulse control disorder/ or exp perception disorder/ or exp psychosocial disorder/ 1466653

7 suicidal ideation/ 20932

8 ((psychological or psychiatric or mental*) adj2 (disorder* or ill or illness* or health or wellness)).ti,ab,kw. 330816

9 (behavio?r adj2 disorder*).ti,ab,kw. 10471

10 (((anxiety or panic or "obsessive-compulsive" or phobi*) adj1 disorder*) or OCD).ti,ab,kw. 71469

11 ((depressi* or bipolar or mood) adj1 disorder*).ti,ab,kw. 116856

12 ("eating disorder*" or anorexi* or bulimi* or "binge eat* disorder*" or pica or "ruminat* disorder*" or "food intake disorder*" or ARFID).ti,ab,kw. 71265

13 "personality disorder*".ti,ab,kw. 27328

14 ("post traumatic stress disorder*" or PTSD).ti,ab,kw. 38746

15 ("psychotic disorder*" or schizophreni*).ti,ab,kw. 173349

16 ((suicid* or selfharm* or "self harm*") adj2 (idea* or thought*)).ti,ab,kw. 18161

17 or/6-16 1642732

18 telemedicine/ or teleconsultation/ or telehealth/ 38753

19 (telecare or telecollaborat* or teleconference* or telehealth or teleguide*or telediagnos* or telemed* or telemonitor* or telepresence* or "telemental health" or telepsychotherap* or ((skype or facetime or zoom or internet or web or online or web or video or distan* or remote*) adj2 (deliver* or conferenc* or call*)) or ehealth* or tele care or tele collaborat* or tele conference* or tele health or tele guide* or tele diagnos* or tele med* or tele monitor* or tele presence* or "tele mental health" or "tele psychotherap*" or (teletherap* not (x-ray or radiat* or cobalt or gamma* or cesium))).mp. 50049

20 ((distan* or remote* or video* or virtual* or tele or web or internet or online or technology or "text messag*") adj4 (psychotherap* or "mental health" or CBT or (cognitive adj2 "behavio?r* therap*"))).mp. 3592

21 telephone/ 37271

22 ((telephone* or telephoning or phone* or phoning) adj2 (call* or deliver* or psychotherap*)).mp. 14992

23 18 or 19 or 20 or 21 or 22 103671

24 5 and 17 and 23 2940

25 (note* or comment* or editorial* or news* or opinion or letter).pt. 2586608

26 24 not 25 2827

27 remove duplicates from 26 2768

**Database: PsycINFO via OVID (1806 - Present)**

**Date of search: July 20 2020**

1 psychotherapy/ 52941

2 exp cognitive behavior therapy/ 21585

3 (psychotherap* or "psycho-therap*").ti,ab,tw. 122199

4 (cognitive adj2 "behavio?r* therap*").ti,ab,tw. 25814

5 or/1-4 155753

6 mental disorders/ or exp affective disorders/ or exp anxiety disorders/ or exp bipolar disorder/ or borderline states/ or exp chronic mental illness/ or exp dissociative disorders/ or exp eating disorders/ or gender dysphoria/ or exp neurosis/ or exp paraphilias/ or exp personality disorders/ or exp psychosis/ or exp sleep wake disorders/ or exp somatoform disorders/ or exp "stress and trauma related disorders"/ or exp "substance related and addictive disorders"/ or thought disturbances/ or confabulation/ or delusions/ or "fantasies (thought disturbances)"/ or "fragmentation (schizophrenia)"/ or judgment disturbances/ or magical thinking/ or obsessions/ or perseveration/ or exp major depression/ or exp obsessive compulsive disorder/ or exp hoarding disorder/ or exp phobias/ or exp mania/ or exp acute psychosis/ or exp alcoholic psychosis/ or exp alcoholic hallucinosis/ or exp childhood psychosis/ or exp "paranoia (psychosis)"/ or exp schizophrenia/ or exp parasomnias/ or exp conversion disorder/ or exp factitious disorders/ or exp attachment disorders/ or exp posttraumatic stress disorder/ or exp nonsubstance related addictions/ or exp "substance use disorder"/ or exp addiction/ or exp "alcohol use disorder"/ or exp "opioid use disorder"/ or exp alcohol withdrawal/ or exp alcoholic psychosis/ or exp alcohol abuse/ 631442

7 suicidal ideation/ 9169

8 ((psychological or psychiatric or mental*) adj2 (disorder* or ill or illness* or health or wellness)).ti,ab,tw. 318930

9 (behavio?r adj2 disorder*).ti,ab,tw. 10012

10 (((anxiety or panic or "obsessive-compulsive" or phobi*) adj1 disorder*) or OCD).ti,ab,tw. 56780

11 ((depressi* or bipolar or mood) adj1 disorder*).ti,ab,tw. 72528

12 ("eating disorder*" or anorexi* or bulimi* or "binge eat* disorder*" or pica or "ruminat* disorder*" or "food intake disorder*" or ARFID).ti,ab,tw. 39099

13 "personality disorder*".ti,ab,tw. 33739

14 ("post traumatic stress disorder*" or PTSD).ti,ab,tw. 37154

15 ("psychotic disorder*" or schizophreni*).ti,ab,tw. 130243

16 ((suicid* or selfharm* or "self harm*") adj2 (idea* or thought*)).ti,ab,tw. 14810

17 or/6-16 863457

18 exp telemedicine/ or exp teleconferencing/ or computer assisted therapy/ or electronic health services/ or computer mediated communication/ or online therapy/ 15547

19 (telecare or telecollaborat* or teleconference* or telehealth or teleguide*or telediagnos* or telemed* or telemonitor* or telepresence* or "telemental health" or telepsychotherap* or ((skype or facetime or zoom or internet or web or online or web or video or distan* or remote*) adj2 (deliver* or conferenc* or call*)) or ehealth* or tele care or tele collaborat* or tele conference* or tele health or tele guide* or tele diagnos* or tele med* or tele monitor* or tele presence* or "tele mental health" or "tele psychotherap*" or (teletherap* not (x-ray or radiat* or cobalt or gamma* or cesium))).mp. 11489

20 ((distan* or remote* or video* or virtual* or tele or web or internet or online or technology or "text messag*") adj4 (psychotherap* or "mental health" or CBT or (cognitive adj2 "behavio?r* therap*"))).mp. 3949

21 ((telephone* or telephoning or phone* or phoning) adj2 (call* or deliver* or psychotherap*)).mp. 3459

22 18 or 19 or 20 or 21 25951

23 5 and 17 and 22 2322

24 (book or "edited book" or "authored book").pt. 487485

25 23 not 24 2093

26 remove duplicates from 25 2092

**Database: Scopus via Elsevier (1976 - Present)**

**Date of search: July 10 2020**

( TITLE-ABS-KEY ( ( psychotherap* OR "psycho-therap*" OR ( cognitive W/2 ( "behavior* therap*" OR "behaviour therap*" ) ) OR cbt ) ) ) AND ( ( TITLE-ABS-KEY ( ( psychological OR psychiatric OR mental* ) W/2 ( disorder* OR ill OR illness* OR health OR wellness ) ) ) OR ( TITLE-ABS-KEY ( behavio?r W/2 disorder* ) ) OR ( TITLE-ABS-KEY ( ( ( anxiety OR panic OR "obsessive-compulsive" OR phobi* ) W/1 disorder* ) OR ocd ) ) OR ( TITLE-ABS-KEY ( ( depressi* OR bipolar OR mood ) W/1 disorder* ) ) OR ( TITLE-ABS-KEY ( "eating disorder*" OR anorexi* OR bulimi* OR "binge eat* disorder*" OR pica OR "ruminat* disorder*" OR "food intake disorder*" OR arfid ) ) OR ( TITLE-ABS-KEY ( "personality disorder*" ) ) OR ( TITLE-ABS-KEY ( "post traumatic stress disorder*" OR ptsd ) ) OR ( TITLE-ABS-KEY ( "psychotic disorder*" OR schizophreni* ) ) OR ( TITLE-ABS-KEY ( ( suicid* OR selfharm* OR "self harm*" ) W/2 ( idea* OR thought* ) ) ) ) AND ( ( ( TITLE-ABS-KEY ( telecare OR telecollaborat* OR teleconferenc* OR telehealth OR teleguide* OR telediagnos* OR telemed* OR telemonitor* OR telepresence* OR "telemental health" OR telepsychotherap* OR ( ( skype OR facetime OR zoom OR internet OR web OR online OR web OR video OR distan* OR remote* ) W/2 ( deliver* OR conferenc* OR call* ) ) OR ehealth* OR "tele care" OR "tele collaborat*" OR "tele conferenc*" OR "tele health" OR "tele guide*" OR "tele diagnos*" OR "tele med*" OR "tele monitor*" OR "tele presence*" OR "tele mental health" OR "tele psychotherap*" OR ( teletherap* AND NOT ( x-ray OR radiat* OR cobalt OR gamma* OR cesium ) ) ) ) OR ( TITLE-ABS-KEY ( ( distan* OR remote* OR video* OR virtual* OR tele OR web OR internet OR online OR technology OR "text messag*" ) W/2 ( psychotherap* OR "mental health" OR "cognitive behavior* therap*" OR "cognitive behaviour* therap*" OR cbt ) ) ) OR ( TITLE-ABS-KEY ( ( telephone* OR telephoning OR phone* OR phoning ) W/2 ( call* OR deliver* OR psychotherap* ) ) ) ) ) AND ( EXCLUDE ( DOCTYPE , "cp" ) OR EXCLUDE ( DOCTYPE , "ed" ) OR EXCLUDE ( DOCTYPE , "ch" ) OR EXCLUDE ( DOCTYPE , "no" ) OR EXCLUDE ( DOCTYPE , "le" ) OR EXCLUDE ( DOCTYPE , "bk" ) OR EXCLUDE ( DOCTYPE , "sh" ) OR EXCLUDE ( DOCTYPE , "er" ) OR EXCLUDE ( DOCTYPE , "Undefined" ) )

Results: 2146

**Database: Web of Science, Core Collection** via Clarivate**

**Date of search: July 10 2020**

Set Number Number of Resuts Search Terms

# 1 103,162 TS=( psychotherap* OR "psycho-therap*" OR ( cognitive NEAR/2 ( "behavior* therap*" OR "behaviour* therap*" ) ) )

# 2 357,906 TOPIC: (( psychological OR psychiatric OR mental* ) NEAR/2 ( disorder* OR ill OR illness* OR health OR wellness ) )

# 3 2,262 TOPIC: (( behavio?r NEAR/2 disorder* ) )

# 4 76,557 TOPIC: (( anxiety OR panic OR "obsessive-compulsive" OR phobi* ) NEAR/1 disorder*)

# 5 11,893 TOPIC: (ocd)

# 6 118,028 TOPIC: (( depressi* OR bipolar OR mood ) NEAR/1 disorder*)

# 7 331,214 TOPIC: ("eating disorder*" OR anorexi* OR bulimi* OR "binge eat* disorder*" OR pica OR "ruminat* disorder*" OR "food intake disorder*" OR arfid OR "personality disorder*" OR "post traumatic stress disorder*" OR ptsd OR "psychotic disorder*" OR schizophreni*)

# 8 16,433 TOPIC: (( ( suicid* OR selfharm* OR "self harm*" ) NEAR/2 ( idea* OR thought* ) ))

# 9 113,489 TOPIC: ("diagnostic and statistical manual" OR "international classification of diseases" OR dsm OR icd)

# 10 812,149 #9 OR #8 OR #7 OR #6 OR #5 OR #4 OR #3 OR #2

# 11 63,688 TS=(telecare OR telecollaborat* OR teleconferenc* OR telehealth OR teleguide* OR telediagnos* OR telemed* OR telemonitor* OR telepresence* OR "telemental health" OR telepsychotherap* OR ((skype OR facetime OR zoom OR internet OR web OR online OR web OR video OR distan* OR remote*) NEAR/2 (deliver* OR conferenc* OR call*) ) OR ehealth* OR "tele care" OR "tele collaborat*" OR "tele conferenc*" OR "tele health" OR "tele guide*" OR "tele diagnos*" OR "tele med*" OR "tele monitor*" OR "tele presence*" OR "tele mental health" OR "tele psychotherapy*" )

# 12 4,228 TS=((distan* or remote* or video* or virtual* or tele or web or internet or online or technology or "text messag*") NEAR/4 (psychotherap* or "mental health" or CBT or "cognitive behavior* therap*" or "cognitive behaviour* therap*") )

# 13 10,443 TS=( ( telephone* OR telephoning OR phone* OR phoning ) NEAR/2 ( call* OR deliver* OR psychotherap* ) )

# 14 76,569 #13 OR #12 OR #11

# 15 2,072 #14 AND #10 AND #1

# 16 1,972 "#14 AND #10 AND #1 Refined by: DOCUMENT TYPES: ( ARTICLE OR REVIEW )

**Database: Cochrane Library via Wiley (1992 - Present)**

**Date of search: July 10 2020**

#1 (psychotherap* or "psycho-therap*"):ti,ab,kw 13896

#2 (cognitive NEAR/2 behavio?r* therap*):ti,ab,kw 19245

#3 #1 or #2 29430

#4 ((psychological or psychiatric or mental*) NEAR/2 (disorder* or ill or illness* or health or wellness)):ti,ab,kw 36142

#5 (behavio?r NEAR/2 disorder*):ti,ab,kw 3231

#6 (((anxiety or panic or obsessive-compulsive or phobi*) NEAR/1 disorder*) or OCD):ti,ab,kw 15608

#7 ((depressi* or bipolar or mood) NEAR/1 disorder*):ti,ab,kw 24299

#8 ("eating disorder*" or anorexi* or bulimi* or "binge eat* disorder*" or pica or "ruminat* disorder*" or "food intake disorder*" or ARFID):ti,ab,kw 8044

#9 personality disorder*:ti,ab,kw 2226

#10 ("post traumatic stress disorder*" or PTSD):ti,ab,kw 4932

#11 ("psychotic disorder*" or schizophreni*):ti,ab,kw 18103

#12 ((suicid* or selfharm* or "self harm*") NEAR/2 (idea* or thought*)):ti,ab,kw 2771

#13 {OR #4-#12} 93621

#14 (telecare or telecollaborat* or teleconference* or telehealth or teleguide*or telediagnos* or telemed* or telemonitor* or telepresence* or "telemental health" or telepsychotherap* or ((skype or facetime or zoom or internet or web or online or web or video or distan* or remote*) NEAR/2 (deliver* or conferenc* or call*)) or ehealth* or tele care or tele collaborat* or tele conference* or tele health or tele guide* or tele diagnos* or tele med* or tele monitor* or tele presence* or "tele mental health" or "tele psychotherap*" or (teletherap* NOT (x-ray or radiat* or cobalt or gamma* or cesium))):ti,ab,kw 9619

#15 ((distan* or remote* or video* or virtual* or tele or web or internet or online or technology or "text messag*") NEAR/4 (psychotherap* or "mental health" or CBT or (cognitive NEAR/2 "behavio?r* therap*"))):ti,ab,kw 1377

#16 ((telephone* or telephoning or phone* or phoning) NEAR/2 (call* or deliver* or psychotherap*)):ti,ab,kw 7810

#17 #14 or #15 or #16 17603

#18 #3 and #13 and #17 1319

**Part 2: Additional tables not included in manuscript**

**Table S1:** Country of publication

| Country of publication | N (%) of references (out of total 48) |
| --- | --- |
|  |  |
| **USA** | 31 (64.6%)  Brenes 2012, 2015, 2017; Celano 2020; Dobkin 2020; Egede 2015; Fann 2015; Frueh 2007; Goetter 2014; Gros 2011; Heffner 2015; Junkins 2020; Kirkness 2017; Liu 2020; Maieritsch 2016; Mitchell 2008; Mohr 2011, 2012; Morland 2010, 2011, 2014, 2015, 2019; Olden 2017; Stecker 2014; Tuerk 2010; Valentine 2020; Wierwille 2016; Yuen 2013, 2015; Ziemba 2014 |
| **Canada** | 9 (18.8%)  Bouchard 2000, 2004, 2020; Dennis 2020; Germain 2009, 2010; Marchand 2011; Taylor 2003; Watts 2020 |
| **Australia** | 4 (8.3%)  Dunstan 2012; Griffiths 2006; Lawn 2019; Stubbings 2013 |
| **Japan** | 2 (4.2%)  Hamatani 2019; Matsumoto 2018 |
| **Spain** | 1 (2.1%)  De las Cuevas 2006 |
| **Norway** | 1 (2.1%)  Vogel 2014 |

**Table S2:** Year of publication

| Year of Publication | N (%) of References (out of total 48) |
| --- | --- |
|  |  |
| **Years (2000-2005)** | 3 (6.3%)  Bouchard 2000, 2004; Taylor 2003 |
| **Years (2006-2010)** | 8 (16.7%)  De las Cuevas 2006; Frueh 2007; Germain 2009, 2010; Griffiths 2006; Mitchell 2008; Morland 2010; Tuerk 2010 |
| **Years (2011-2015)** | 21 (43.8%)  Brenes 2012, 2015; Dunstan 2012; Egede 2015; Fann 2015; Goetter 2014; Gros 2011; Heffner 2015; Marchand 2011; Mohr 2011, 2012; Morland 2011, 2014, 2015; Stecker 2014; Vogel 2014; Yuen 2013, 2015; Ziemba 2014 |
| **Years (2016-2020)** | 16 (33.3%)  Bouchard 2020; Brenes 2017; Celano 2020; Dennis 2020; Hamatani 2019; Junkins 2020; Lawn 2019; Liu 2020; Kirkness 2017; Maieritsch 2016; Matsumoto 2018; Morland 2019; Olden 2017; Valentine 2020; Watts 2020; Wierwille 2016 |

**Table S3:** Research study design

| Study Design | N (%) of References (out of total 48) |
| --- | --- |
|  |  |
| **RCT** | 32 (66.7%)  Bouchard 2000, 2004; Brenes 2012, 2015, 2017; Celano 2020; De las Cuevas 2006; Dennis 2020; Dobkin 2020; Dunstan 2012; Edge 2015; Fann 2015; Frueh 2007; Junkins 2020; Kirkness 2017; Liu 2020; Maieritsch 2016; Mitchell 2008; Mohr 2011, 2012; Morland 2010, 2011, 2014, 2015, 2019; Olden 2017; Stecker 2014; Stubbings 2013; Vogel 2014; Watts 2020; Yuen 2015; Ziemba 2014 |
| **Non-randomized controlled trial** | 8 (16.7%)  Bouchard 2020; Germain 2009, 2010; Gros 2011; Heffner 2015; Marchand 2011; Tuerk 2010; Valentine 2020 |
| **Pre/Post-test (No control group)** | 6 (12.5%)  Goetter 2014; Griffiths 2006; Hamatani 2019; Lawn 2019; Matsumoto 2018; Yuen 2013 |
| **Other** | 2 (4.2%)  Taylor 2003; Wierwille 2016 |
| **Qualitative** | 0 (0%) |
| **Mixed (quantitative & qualitative)** | 2 (4.2%)  Lawn 2019, Junkins 2020 |

**Table S4:** Population type (military vs civilian)

| Population Type | N (%) References (out of total 48) |
| --- | --- |
|  |  |
| **Military** | 16 (33.3%)  Egede 2015; Frueh 2007; Gros 2011; Liu 2020; Maieritsch 2016; Mohr 2011; Morland 2010, 2011, 2014, 2019; Stecker 2014; Tuerk 2010; Valentine 2020; Wierwille 2016; Yuen 2015; Ziemba 2014 |
| **Civilian** | 32 (66.7%)  Bouchard 2000, 2004, 2020; Brenes 2012, 2015, 2017; Celano 2020; De las Cuevas 2006; Dennis 2020; Dobkin 2020; Dunstan 2012; Fan 2015; Germain 2009, 2010; Goetter 2014; Griffiths 2006; Hamatani 2019; Heffner 2015; Junkins 2020; Kirkness 2017; Lawn 2019; Marchand 2011; Matsumoto 2018; Mitchell 2008; Mohr 2012; Morland 2015; Olden 2017; Stubbings 2013; Taylor 2003; Vogel 2014; Watts 2020; Yuen 2013 |

**Table S5:** DSM/ICD diagnosis

| DSM/ICD Diagnosis | N (%) of References (out of total 48) |
| --- | --- |
|  |  |
| **Posttraumatic stress disorder (PTSD)** | 19 (40.0%)  Frueh 2007; Germain 2009, 2010; Gros 2011; Liu 2020; Maieritsch 2016; Marchand 2011; Morland 2010, 2011, 2014, 2015, 2019; Olden 2017; Stecker 2014; Tuerk 2010; Valentine 2020; Wierwille 2016; Yuen 2015; Ziemba 2014 |
| **Panic disorder (PD)** | 2 (4.2%)  Benes 2012; Matsumoto 2018 |
| **Panic disorder with agoraphobia (PDA)** | 4 (6.3%)  Bouchard 2000, 2004, 2020; Griffiths 2006; |
| **Anxiety disorder** | 10 (20.8%)  Brenes 2012, 2015, 2017; Dunstan 2012; Griffiths 2006; Lawn 2019; Matsumoto 2018;Stubbings 2013; Watts 2020; Yuen 2013 |
| **Depressive disorder** | 11 (22.9%)  Dennis 2020; Dobkin 2020; Egede 2015; Fann 2015; Griffiths 2006; Junkins 2020; Kirkness 2017; Lawn 2019; Mohr 2011, 2012; Olden 2017; Stubbings 2013 |
| **Mixed anxiety-depressive disorder** | 2 (4.2%)  Dunstan 2012; Griffiths 2006 |
| **Obsessive-compulsive disorder (OCD)** | 4 (8.3%)  Goetter 2014; Matsumoto 2018; Taylor 2003; Vogel 2014 |
| **Eating disorder** | 2 (4.2%)  Hamatani 2019; Mitchell 2008; |
| **Bipolar disorder** | 2 (4.2%)  Celano 2020; Heffner 2015 |
| **Neurotic, stress-related and somatoform disorders** | 1 (2.1%)  De Las Cuevas 2006 |

**Table S6:** Intervention characteristics

| Intervention Characteristic | N (%) of References (out of total 48) |
| --- | --- |
|  |  |
| **Mode of Delivery** |  |
| Video | 31 (64.6%)  Bouchard 2000, 2004, 2020; De las Cuevas 2006; Dunstan 2012; Egede 2015; Frueh 2007; Germain 2009, 2010; Goetter 2014; Griffiths 2006; Gros 2011; Hamatani 2019; Junkins 2020; Liu 2020; Maieritsch 2016; Marchand 2011; Matsumoto 2018; Morland 2010, 2011, 2014, 2015; Olden 2017; Stubbings 2013; Tuerk 2010; Valentine 2020; Vogel 2014; Watts 2020; Wierwille 2016; Yuen 2013, 2015 |
| Phone | 15 (31.3%)  Brenes 2012, 2015, 2017; Celano 2020; Dennis 2020; Dobkin 2020; Fann 2015; Heffner 2015; Kirkness 2017; Lawn 2019; Mohr 2011, 2012; Stecker 2014; Taylor 2003; Vogel 2014 |
| Both (Video & Phone) | 1 (2.1%)  Vogel 2014 |
| Unspecified (Ex. “telemedicine”) | 3 (6.3%)  Mitchell 2008; Morland 2019; Ziemba 2014 |
| **Group or 1-1** |  |
| Group | 4 (8.3%)  Frueh 2007; Morland 2010, 2011; 2014 |
| 1-1 | 44 (91.7%)  Bouchard 2000, 2004, 2020; Brenes 2012, 2015, 2017; Celano 2020; De las Cuevas 2006; Dennis 2020; Dobkin 2020; Dunstan 2012; Egede 2015; Fann 2015; Germain 2009, 2010; Goetter 2014; Griffiths 2006; Gros 2011; Hamatani 2019; Heffner 2015; Junkins 2020; Kirkness 2017; Lawn 2019; Liu 2020; Maieritsch 2016; Marchand 2011; Matsumoto 2018; Mitchell 2008; Mohr 2011, 2012; Morland 2015, 2019; Olden 2017; Stecker 2014; Stubbings 2013; Taylor 2003; Tuerk 2010; Valentine 2020; Vogel 2014; Watts 2020; Wierwille 2016; Yuen 2013, 2015; Ziemba 2014 |
| **Type of Therapy** |  |
| Cognitive behavioural therapy (CBT) | 27 (56.3%)  Bouchard 2000, 2004, 2020; Brenes 2012, 2015, 2017; De las Cuevas 2006; Dobkin 2020; Dunstan 2012; Fann 2015; Frueh 2007; Germain 2009, 2010; Hamatani 2019; Junkins 2020; Lawn 2019; Marchand 2011; Matsumoto 2018; Mitchell 2008; Mohr 2011, 2012; Stecker 2014; Stubbings 2013; Taylor 2003; Watts 2020; Wierwille 2016; Ziemba 2014 |
| Cognitive processing therapy (CPT) | 6 (12.5%)  Liu 2020; Maieritsch 2016; Morland 2011, 2014, 2015; Valentine 2020 |
| Exposure therapy or Prolonged Exposure (PE) | 7 (14.6%)  Gros 2011; Morland 2019; Olden 2017; Tuerk 2010; Valentine 2020; Wierwille 2016; Yuen 2015 |
| Exposure and ritual prevention (ERP) | 4 (8.3%)  Goetter 2014; Griffiths 2006; Taylor 2003; Vogel 2014 |
| Acceptance and commitment therapy | 1 (2.1%)  Heffner 2015 |
| Positive psychology (PP) | 1 (2.1%)  Celano 2020 |
| Interpersonal therapy | 2 (4.2%)  Dennis 2020; Dunstan 2012 |
| Behavioral activation | 1 (2.1%)  Egede 2015 |
| Anger management therapy | 1 (2.1%)  Morland 2010 |
| Brief psychosocial behavioral intervention | 1 (2.1%)  Kirkness 2017 |
| Acceptance Based Behavior Therapy for Social Anxiety Disorder (ABBT for SAD) | 1 (2.1%)  Yuen 2013 |

**Table S7:** Study outcomes

| Outcome | N (%) of References (out of total 48) |
| --- | --- |
|  |  |
| **Remote delivery at least as good as in person** | 32 (66.7%)  Bouchard 2000, 2004, 2020; De las Cuevas 2006; Dennis 2020; Dunstan 2012; Egede 2015; Fann 2015; Frueh 2007; Germain 2009, 2010; Hamatani 2019; Heffner 2015; Kirkness 2017; Lawn 2019; Liu 2020; Maieritsch 2016; Marchand 2011; Mitchell 2008; Mohr 2012; Morland 2010, 2011, 2014, 2015, 2019; Stubbings 2013; Tuerk 2010; Vogel 2014; Watts 2020; Yuen 2013, 2015; Ziemba 2014 |
| **Remote delivery not compared to an in person comparator** | 12 (25.0%)  Brenes 2012, 2015, 2017; Celano 2020; Dobkin 2020; Goetter 2014; Griffiths 2006; Junkins 2020; Matsumoto 2018; Olden 2017; Stecker 2014; Taylor 2003 |
| **Remote delivery not as effective/efficacious as in person** | 4 (8.3%)  Gros 2011; Mohr 2011; Valentine 2020; Wierwille 2016 |
