# Supplementary material for: Synchronous Web-Based Psychotherapy for Mental Disorders From a Health Quality Perspective: Scoping Review
Source: J Med Internet Res. 2023 Nov 3;25:e40710. doi: 10.2196/40710 (PMC10656669; doi:10.2196/40710)
Supplement: Multimedia Appendix 3 [file jmir_v25i1e40710_app3.docx]

**Multimedia Appendix 3**

**Table S1:** Online psychotherapy scoping review study characteristics table

| Study type | Study population | Sample size | Mean age (SD) | N (%) male | Mode of Delivery (Phone or Video) | Group or 1-1 | Author, year (Country) |
| --- | --- | --- | --- | --- | --- | --- | --- |
|  |  |  |  |  |  |  |  |
| **Cognitive Behavioral Therapy (n=27)** | | | | | |  |  |
| RCT | Adults with PDA | 8 | 30 | 37.5% | Video | 1-1 | Bouchard et al., 2000 (Canada) |
| RCT | Adults with PDA | 21  Local site: N = 10  Remote site: N = 11 | 37.9  Local site: 37.1  Remote site: 38.8 | 28.6 | Video | 1-1 | Bouchard et al., 2004 (Canada) |
| RCT | Adults aged 60 years and older with GAD, PD, or ADNOS | 60  CBT-T group n = 30  Information-only group n = 30 | 69.2 (7.1)  CBT-T group: 68.8 (7.3)  Information-only group: 69.5 (6.9) | CBT-T group: 16.7% Information-only group: 16.7% | Phone | 1-1 | Brenes et al., 2012 (USA) |
| RCT | Rural adults aged 60 years and older with GAD | 141  CBT-T group n = 70  NST-T group n = 71 | Age groups: 60–64: 46.8%  65–69: 27.0%  70–74: 13.5%  ≥75: 12.8% | 18.4% | Phone | 1-1 | Brenes et al., 2015 (USA) |
| RCT | Rural adults aged 60 years and older with GAD | 141  CBT N=70  NST N=71 | 66.8 (6.2) | 18.4% | Phone | 1-1 | Brenes et al., 2017(USA) |
| RCT | Adult outpatients with mental and behavioral disorders due to psychoactive substance abuse;  schizophrenia, schizotypal, and delusional disorders;  mood disorders;  neurotic, stress-related and somatoform disorders; or disorders of the adult personality and behavior | 140 total:  VC N=70; FTF N=70 | NR | 33.6% total  VC: 31.4%; FTF: 35.7% | Video | 1-1 | De las Cuevas et al., 2006 (Spain) |
| RCT | Adults aged 35–85 years with PD and depressive disorder | N=72;  CBT N=37; TAU N=35 | 65.22 (9.63) | 48.61% | Phone | 1-1 | Dobkin, 2020 (USA) |
| RCT | Adults with a mood or anxiety disorder | N = 6 ; VC n = 3; FTF n = 3 | 34.33 (16.11) | 2 (33.3%) | Video | 1-1 | Dunstan, 2012 (Australia) |
| RCT | Adults with MDD who were hospitalized within the past 10 years for a complicated mild to severe TBI | Telephone CBT = 40;  In-person CBT = 18;  UC = 42 | All: 45.8 (13.3)  CBT: 45.4 (14.1) UC: 46.3 (12.4) | Male: CBT= 34 (59%) UC: 29 (69%) | Phone | 1-1 | Fann et al., 2015 (USA) |
| RCT | Male veterans with PTSD | Total n = 38  Same-room group n = 21  Telepsychiatry group n = 17 | Same-room group: 56 (5)  Telepsychiatry group: 55 (5) | 100% | Video | Group | Frueh, 2007(USA) |
| RCT | African American HIV+ females aged 19 years and older with depression | Total N = 22; CBT group N = 11; ISP group N = 11 | Overall 45.8 (11.8); CBT-AD intervention 48 (11.3); ISP intervention 43 (12) | 0% | Video | 1-1 | Junkins et al., 2020 (USA) |
| RCT | Adults with BN (purging or non-purging subtype) or eating disorder not otherwise specified | n=128; FTF n=66; telemedicine n=62 | FTF group = 29.6 (10.9); Telemedicine group: 28.4 (10.4) | FTF group: male 3%; Telemedicine group: male 0% | telemedicine system (TV-CBT) | 1-1 | Mitchell et al., 2008 (USA) |
| RCT | Veterans with MDD | 85 enrolled; 41 assigned to T-CBT; 44 assigned to TAU | 55.9 (10.59) | 90.6% | Phone | 1-1 | Mohr et al., 2011 (USA) |
| RCT | Adults with MDD | Total n=325; FTF group n=162; telephone group n=163 | FTF group: 47.5 (13.5); Telephone group: 47.8 (12.6) | FTF group: male= 21.6%  Telephone group: male= 23.4% | Phone | 1-1 | Mohr et al., 2012 (USA) |
| RCT | Veterans with PTSD | Total n=274; Intervention n=123; control n=151 | Mean age total = 29; Intervention Group = 28.3 (5.3); Control Group = 30.2 (6.9) | Total: 87.2%;Intervention Group: male 84.3%; Control Group: 90.1% | Phone | 1-1 | Stecker, 2014 (USA) |
| RCT | Adults with a DSM-IV axis one disorder living in Perth, Western Australia | n= 26 participants;  VC = 14;  In-Person= 12 | 30 (11) | Total 42% (11)  VC: 42.9%; In-Person: 41.7% | Video | 1-1 | Stubbings et al., 2013 (Australia) |
| RCT | Adults aged between 18 and 75 with GAD | Total n=115; VC group n= 50; conventional psychotherapy group n=65 | Telepsychotherapy via  VTC: 43 (15.00)  Conventional psychotherapy: 40 (16.00) | NR | Video | 1-1 | Watts et al., 2020 (Canada) |
| RCT | Veterans with PTSD | n=18 enrolled; n=13 completed; n=6 FTF; n=7 telemedicine | NR | NR | telemedicine (could be over the phone, but not specified) | 1-1 | Ziemba, 2014 (USA) |
| Non-randomized controlled trial | Adults with PDA | 71  Video= 40  FTF = 31 | Video: 34.9 (10.45)  FTF: 36.90 (11.60) | Video: 15%  FTF: 19% | Video | 1-1 | Bouchard et al., 2020 (Canada) |
| Non-randomized controlled trial | Adults between 18 and 65 years with PTSD | VC (n=16)  FTF (n=32) | VC = 43 (11)  FTF = 42 (12) | VC = 6 (37.5%)  FTF = M:13 (41%) | Video | 1-1 | Germain, 2009 (Canada) |
| Nonrandomized controlled trial | Adults with PTSD | 46 total. 29 FTF; 17 VC | 42 | Overall: 41.3%. VC group: 35.3%; FTF group: 44.8%. | Video | 1-1 | Germain et al., 2010 (Canada) |
| Non-randomized controlled trial | Adults with PTSD | n=68 (n=44 VC; n=22 FTF) | 42.1 (12.1) | 36% | Video | 1-1 | Marchand, 2011 (Canada) |
| Non-randomized controlled trial | Veterans with PTSD | Telehealth n =85; outpatient n=136 | Telehealth 47.13 (15.39); outpatient 46.35 (38.01) | NR | Video | 1-1 | Wierwille et al., 2016 (USA) |
| Pre/Post-test (No control group) | Females aged 18 to 65 years with BN or binge-eating disorder | N = 7 | 31.9 (7.9) | 0, 0% | Video | 1-1 | Hamatani, 2019 (Japan) |
| Pre/Post-test (No control group) | Adults with MDD or anxiety disorder | Total N = 680; Completers N = 427; Non-completers N = 253 | Completers: 55.5 (15.6) Non-completers: 52.1 (16.3) | Completers: 59.5%  Non-completers: 40.5% | Phone | 1-1 | Lawn et al., 2019 (Australia) |
| Pre/Post-test (No control group) | Adults aged 19 to 65 years with OCD, PD, or SAD | N=30, 1 drop out | 35.4 (9.2) | 20% | Video | 1-1 | Matsumoto, 2018 (Japan) |
| Cohort design, used pre/post test | Adults with OCD | N = 33, completed;  N = 26  Delayed treatment n = 15 (completed);  Immediate treatment n = 11 (completed) | 38 (12) | 24% | Phone, use FTF for screening | 1-1 | Taylor et al., 2003 (Canada) |
| **Exposure Therapy or Prolonged Exposure (n=7)** | | | | | |  |  |
| RCT | Veterans with PTSD | Total N = 175 IHIP n = 58 HBT n = 58 OBT n = 59 | 46.5 (14.11) | 74.9% | Unspecified | 1-1 | Morland et al., 2019 (USA) |
| RCT | Adults aged 18–70 years who were survivors of trauma resulting from working in an occupation at risk for PTSD (e.g. disaster workers, firefighters, police officers, military service workers, reservists, and veterans) with PTSD | 11 | 42.82 (13.53) | 81.8% | Video | 1-1 | Olden et al., 2017 (USA) |
| RCT | Veterans and military personnel with PTSD | 52 | 43.98 (15.18) | 98.1% | Video | 1-1 | Yuen, 2015 (USA) |
| Non-randomized controlled trial | Veterans with PTSD | N = 89; Telehealth n=62; In person n = 27 | Telehealth group: 45.1 (15.0), In-person group: 45.2 (16.0) | Telehealth group: 93.5%; In-person group: 88.9% | Video | 1-1 | Gros, 2011 (USA) |
| Non-randomized controlled trial | Veterans with combat-related PTSD | PE Live n = 35  PE via telehealth n = 12 | 39 (16) | 94% | Video, use FTF for screening | 1-1 | Tuerk et al., 2010 (USA) |
| Non-randomized controlled trial | Veterans with PTSD | n = 171 (18.1% CVT-enrolled)  CVT n = 32  FTF n = 140 | 44.4 (11.6) | 26.5% | Video | 1-1 | Valentine et al., 2020 (USA) |
| Non-randomized controlled trial | Veterans with PTSD | Telehealth n =85; outpatient n=136 | Telehealth 47.13 (15.39); outpatient 46.35 (38.01) | NR | Video | 1-1 | Wierwille et al., 2016 (USA) |
| **Cognitive Processing Therapy (n=6)** | | | | | |  |  |
| RCT | Veterans with PTSD | Total n = 207, in person group = 104, VC group = 103 | 48.4 (14.1) | 77.4% | Video | 1-1 | Liu L. et al., 2020 (USA) |
| RCT | Veterans with PTSD | 90 (divided 45 in each group) | 30.93 (6.05) | 93% | Video | 1-1 | Maieritsch et al., 2016 (USA) |
| RCT | Veterans with combat-related PTSD | 13 enrolled, 7 assigned to NP group, 6 assigned to VTC group | The mean age was 48.6 years (14.2; range = 29–61) for the NP and 53.0 years (19.6; range = 28–77) for the VTC conditions. | 100% | Video | Group | Morland et al., 2011 (USA) |
| RCT | Veterans with PTSD | N = 125, In person group = 64, VTC group = 61 | 55.3 (12.5) | 100% | Video | Group | Morland, 2014 (USA) |
| RCT | Veterans with PTSD | Total sample (N = 126)  In-Person group n = 63;  VTC group n = 63 | 46.4 (11.9) | 0% | Video | 1-1 | Morland, 2015 (USA) |
| Non-randomized controlled trial | Veterans with PTSD | n = 171 (18.1% CVT-enrolled)  CVT n = 32  FTF n = 140 | 44.4 (11.6) | 26.5% | Video | 1-1 | Valentine et al., 2020 (USA) |
| **Exposure and Ritual Prevention (n=4)** | | | | | |  |  |
| RCT | Adults with OCD | n = 10 in VCT group, n = 10 in self-help group; n = 10 in waitlist group | VCT group: 28.8 (9.2); Self-help group: 29.8 (10.3); Waitlist group: 40.7 (11.1) | 40% VCT; 50% self-help; 30% waitlist | Video, Phone | 1-1 | Vogel, 2014 (Norway) |
| Pre/Post-test (No control group) | Adults with OCD | N=15 | 32.2 (11.41) | 13.3% | Video | 1-1 | Goetter, 2014 (USA) |
| Pre/Post-test (No control group) | Adults with GAD, PDA, MDD or mixed anxiety and depressive disorder | N=15 | NR | 20% | Video | 1-1 | Griffiths, 2006 (Australia) |
| Cohort design, used pre/post test | Adults with OCD | N = 33, completed n = 26  Delayed treatment n = 15 (completed)  Immediate treatment n = 11 (completed) | 38 (12) | 24% | Phone, use FTF for screening | 1-1 | Taylor et al., 2003 (Canada) |
| **Interpersonal Therapy (n=2)** | | | | | |  |  |
| RCT | Adult women with depression between 2 and 24 weeks postpartum | 241  Control Group = 121  Intervention = 120 | NR | 0% | Phone | 1-1 | Dennis et al., 2020 (Canada) |
| RCT | Adults with a mood or anxiety disorder | N = 6 ; VC n = 3; FTF n = 3 | 34.33 (16.11) | 2 (33.3%) | Video | 1-1 | Dunstan, 2012 (Australia) |
| **Acceptance and Commitment Therapy (n=1)** | | | | | |  |  |
| Non-randomized controlled trial | Adults with bipolar disorder who were daily smokers and motivated to quit in the next  30 days | In-person n=10  Telephone n=6 | In-person: 42.1 (16.1); Telephone: 51.0 (15.4) | In-person: 20%;  Telephone: 17% | Phone | 1-1 | Heffner et al., 2015 (USA) |
| **Acceptance Based Behavior Therapy for Social Anxiety Disorder (n=1)** | | | | | |  |  |
| Pre/Post-test (No control group) | Adults with SAD | n = 24 | 35.0 ( 10.8) | 75% | Video | 1-1 | Yuen et al., 2013 (USA) |
| **Anger Management Therapy (n=1)** | | | | | |  |  |
| RCT | Veterans with PTSD | N = 125, n=64 in-person group, n=61 VTC group (112 participants finished treatment: 57 in-person; 55 VTC) | In-person group mean age: 54.7 (9.7); VTC group mean age: 54.8 (9.3) | 100% | Video | Group | Morland et al., 2010 (USA) |
| **Behavioral Activation (n=1)** | | | | | |  |  |
| RCT | Veterans aged 58 years or older with MDD | N= 241  Telemedicine N=120  Same-room N= 121 | 63.9 (5.1) | 98% | Video | 1-1 | Egede, 2015 (USA) |
| **Brief Psychosocial Behavioral Intervention (n=1)** | | | | | |  |  |
| RCT | People with depression within 4 months of an ischemic or hemorrhagic stroke | Total= 100  Telephone=37  In-person=35 Control=28 | 60 | 50% | Phone | 1-1 | Kirkness et al., 2017 (USA) |
| **Positive Psychology (n=1)** | | | | | |  |  |
| RCT | Adults with bipolar depression | PP = 14  Control = 11 | PP: 42.6 (12.8)  Control: 42.6 (12.8) | 32% | Phone | 1-1 | Celano et al., 2020 (USA) |

**Abbreviations:**

PDA: Panic Disorder with Agoraphobia

GAD: Generalized Anxiety Disorder (GAD)

PD: Panic Disorder (PD)

ADNOS: Anxiety Disorder Not Otherwise Specified

PD: Parkinson’s Disease

SAD: Social Anxiety Disorder

MDD: Major Depressive Disorder

BN: Bulimia Nervosa

PTSD: Posttraumatic Stress Disorder

HIV: Human Immunodeficiency virus

OCD: Obsessive Compulsive Disorder

CBT-T: Cognitive Behavioral Therapy Delivered by Telephone

CVT: Clinical Video Technology

NST-T: Telephone-delivered Nondirective Supportive Therapy

PE: Prolonged Exposure Therapy

PP: Positive Psychology

VCT: Videoconference-assisted Exposure and Response Prevention

VTC: Video Teleconference

RCT: Randomized Control Trial

NR: Not Reported

TAU: Treatment as Usual

TBI: Traumatic Brain Injury

UC: Usual Care

DSM: Diagnostic and Statistical Manual of Mental Disorders

NP: In-person

HBT: Home-based Telehealth

OBT: Office-based Telehealth

IHIP: In-person-in-home

FTF: Face-to-face

VC: Videoconference

**Table S2:** Online psychotherapy scoping review results table

| Acceptability | Accessibility | Appropriateness | Effectiveness | Efficiency | Safety | Author, year (Country) |
| --- | --- | --- | --- | --- | --- | --- |
|  |  |  |  |  |  |  |
| **Cognitive Behavioral Therapy (n=27)** | | | | | |  |
| Yes  (High WAI scores; high rating of perception toward telepsychotherapy on 5-point scale) | No | No | Yes  (significant improvement on all outcome measures - PAS, SE-CPAQ, STAI, DISS) | No | No | Bouchard et al., 2000 (Canada) |
| Yes  (High WAI scores) | No | No | Yes  (Significant improvement in outcome measures - ACQ, BSQ, MI, SE-CPAQ, STAI, BDI, DISS) | No | No | Bouchard et al., 2004 (Canada) |
| Yes  (High WAI scores; high CALPAS scores) | No | No | Yes  (non-inferiority on outcome measures - PAS, MI, BSQ, BDI, ACQ) | No | No | Bouchard et al., 2020 (Canada) |
| Yes  (high satisfaction scores on CSQ-8; high WAI-S scores) | No | No | Yes  (improvement in anxiety symptoms - PSWQ, STAI) | No | No | Brenes et al., 2012 (USA) |
| Yes  (high satisfaction scores on CSQ-8) | No | No | Yes  (improvement in anxiety symptoms - PSWQ, GAD-7, BDI) | No | No | Brenes et al., 2015 (USA) |
| No | No | No | Yes  (improvement in anxiety symptoms - HAM-A, PSWQ, GAD-7, BDI) | No | No | Brenes et al., 2017 (USA) |
| No | No | No | Yes  (improvement in symptoms - CGI, GSI, PSDI, PST) | No | No | De las Cuevas et al., 2006 (Spain) |
| No | No | No | Yes  (significant improvements in outcome measures - HAM-D, CGI, BDI, HAM-A, SF-36, MCS) | No | No | Dobkin et al., 2020 (USA) |
| No | No | No | Yes (significant improvement in DASS-42 scores) | No | No | Dustan, et al., 2012 (Australia) |
| Yes  (high WAI-S scores) | No | No | Yes  (no difference in effectiveness between digital and in-person therapy - HAM-D, SCL-20, PGI) | No | No | Fann et al., 2015 (USA) |
| Yes  (high satisfaction ratings - CPOSS-VA; high scores on SDP) | No | No | Yes  (clinical outcomes similar between groups - LEC, PCL-M, SCL-90-R, GSI, BDI) | No | No | Frueh, 2007 (USA) |
| Yes  (high scores on DCSS; high scores on VT-Q) | No | No | Yes  (primary mental health outcomes similar between groups - MPSS, BDI, BAI, ACF) | No | No | Germain, 2009 (Canada) |
| Yes  (high WAI scores; high DCSS scores; high scores on VT-Q) | No | No | Yes (significant decline in the severity and frequency of PTSD symptoms on MPSS) | No | No | Germain, 2010 (Canada) |
| Yes  (high WAI-SF scores) | No | No | Yes  (ICBT via videoconference effective for treating bulimia nervosa and binge-eating  disorder - reduction in some binge/purge episodes) | No | No | Hamatani, 2019 (Japan) |
| Yes  (high CSQ-8 scores) | No | No | Yes  (decrease in CES-D scores over treatment period) | No | No | Junkins et al., 2020 (USA) |
| No | No | No | Yes  (improvement in PHQ-9 and GAD-7 scores) | No | No | Lawn et al., 2019 (Australia) |
| No | No | No | Yes  (non-inferiority in clinical outcome scores for virtual CBT for PTSD - MPSS, BDI-II, BAI, SF-12) | No | No | Marchand, 2011 (Canada) |
| Yes  (high satisfaction ratings on 7-point Likert scale; high WAI-SF scores) | No | No | Yes  (reductions in symptoms of OCD, panic, and social anxiety; reductions in PHQ-9 and GAD-7 scores) | No | No | Matsumoto, 2018 (Japan) |
| Yes (WAI scores indicate the intervention was acceptable to patients) | No | No | Yes  (reduction in binge purge episodes and BDI scores) | No | No | Mitchell et al., 2008 (USA) |
| No | No | No | Yes  (no differences in HAM-D or PHQ-9 scores or episodes of MD between T-CBT and treatment as usual) | No | No | Mohr et al., 2011 (USA) |
| No | No | No | Yes  (significant improvement in depression symptoms - HAM-D, PHQ-9) | No | Yes  (no suicidal ideation or suicide attempts for either treatment condition) | Mohr et al., 2012 (USA) |
| No | No | No | Yes  (both groups reported reductions in symptoms of both PTSD and  depression during course of trial) Measures: PASS, PCL-M, PHQ-9 | No | No | Stecker, 2014 (USA) |
| Yes  (high WAI-S scores; high CSQ-8 scores; high TSQ scores) | No | No | Yes  (CBT was effective in significantly reducing symptoms of depression, anxiety, and stress - DASS, Q-LES-Q, BDI-II, OCI, HAQ, PSWQ, ASI) | No | No | Stubbings et al., 2013 (Australia) |
| No | No | No | Yes  (scores for interview-administered Dimensional YBOCS, self-  report YBOCS and PI declined significantly over the course of treatment) | No | No | Taylor et al., 2003 (Canada) |
| Yes  (high WAI scores) | No | No | Yes  (clinical and statistical improvement of the participants on ADIS-IV between the beginning and end of the therapy) | No | No | Watts et al., 2020 (Canada) |
| No | No | No | Yes  (Veterans who received CPT and PE treatment in traditional outpatient setting experienced greater PTSD symptom reduction than those who received same treatments in telehealth setting - PCL-S, BDI-2) | No | No | Wierwille et al., 2016 (USA) |
| Yes  ( survey results indicate greater satisfaction for  telemedicine subjects as opposed to those receiving traditional face-  to-face treatment) | No | No | Yes  (reductions in CAPS, HAM-A, and MADRS scores signify improvement of symptoms) | No | No | Ziemba, 2014 (USA) |
| **Exposure Therapy or Prolonged Exposure (n=7)** | | | | | |  |
| No | No | No | Yes  (significant reductions across all measures of symptomatology in telehealth and in-person groups - PCL-M, DASS, BDI-II, IIRS) | No | No | Gros, 2011 (USA) |
| No | No | No | Yes  (On average, all participants experienced improvements across primary and secondary clinical outcomes, independent of treatment modality. Improvements were maintained over time. Measures: BAI, BDI‐II, B-IPF, CAPS-5, PCL-5) | No | Yes  (Neither  participants nor providers reported safety issues, regardless of  modality) | Morland et al., 2019 (USA) |
| Yes  (high CSQ-8 scores; high WAI-SF scores; high TSAS scores) | No | No | Yes  (post-treatment: all study completers demonstrated significant  reductions across clinical measures - CAPS, PCL, BDI) | No | Yes  (No safety issues arose during any of the assessments or treatment sessions) | Olden et al., 2017 (USA) |
| No | No | No | Yes  (Prolonged exposure treatment via telehealth associated with large reductions in symptoms of PTSD and depression for veterans diagnosed with combat-related PTSD - measured via PCL and BDI) | No | Yes  (no instances in the PE telehealth condition where an event representing a threat to  patient safety occurred) | Tuerk et al., 2010 (USA) |
| No | No | No | Yes  (trend of fewer participants fully completing and attriting quicker in CVT; similar trend when considering “minimally  adequate care”) | No | No | Valentine et al., 2020 (USA) |
| No | No | No | Yes  (Veterans who received CPT and PE treatment in traditional outpatient setting experienced greater PTSD symptom reduction than those who received same treatments in telehealth setting - PCL-S, BDI-2) | No | No | Wierwille et al., 2016 (USA) |
| Yes  (high scores on SDP) | No | No | Yes  (Symptoms of PTSD, depression,  and anxiety declined in both treatment modalities. Noninferiority supported for CAPS and anxiety (BAI); results inconclusive for self-reported  PTSD symptoms (PCL) and depression (BDI-II)) | No | No | Yuen, 2015 (USA) |
| **Cognitive Processing Therapy (n=6)** | | | | | |  |
| No | No | No | Yes  (self-reported PTSD, clinician-  rated PTSD, and self-reported depression  instrument scores showed symptom severity decreases - CAPS, PCL-S, PHQ-9) | No | No | Liu L. et al., 2020 (USA) |
| No | No | No | Yes  (decreased BDI, CAPS, PCL scores in telemental health group) | No | No | Maieritsch et al., 2016 (USA) |
| Yes  (high TSAS scores; no difference in GTAS scores between treatment conditions) | No | No | Yes  (differences between CAPS scores at pre-  treatment compared to posttreatment,  and 6-month follow-up) | No | No | Morland et al., 2011 (USA) |
| Yes  (high CPOSS-VA scores; high scores on TSAS; high GTAS scores) | No | No | Yes  (reductions in CAPS scores, no  significant effect for treatment condition  at any time point) | No | No | Morland, 2014 (USA) |
| Yes  (High WAI-SF scores; high CPOSS-VA scores; high TSAS scores) | No | No | Yes  (civilians demonstrated reductions at all time points; veterans demonstrated no reductions in CAPS at any time point) | No | No | Morland, 2015 (USA) |
| No | No | No | Yes  (trend of fewer participants fully completing and attriting quicker in CVT; similar trend when considering “minimally  adequate care”) | No | No | Valentine et al., 2020 (USA) |
| **Exposure and Ritual Prevention (n=4)** | | | | | |  |
| Yes  (high WAI-S scores; high satisfaction scores on CSS; high scores on RTQ) | No | No | Yes  (YBOCS scores changed significantly over time) | No | No | Goetter, 2014 (USA) |
| Yes  (high acceptability rating on Likert-type scale) | No | No | Yes  (significant improvement in MHI scores) | No | No | Griffiths, 2006 (Australia) |
| No | No | No | Yes  (scores for interview-administered Dimensional YBOCS, self-  report YBOCS and PI declined significantly over the course of treatment) | No | No | Taylor et al., 2003 (Canada) |
| Yes  (high WAI scores) | No | No | Yes  (CVT group had greatest change in primary outcome (Y-BOCS) score; secondary outcome measures (VOCI and BDI) did not show significant differences among groups) | No | No | Vogel, 2014 (Norway) |
| **Interpersonal Therapy (n=2)** | | | | | |  |
| Yes  (high scores on satisfaction questionnaire) | No | No | Yes  (improvement in depressive symptoms: EPDS24, STAI, DAS, ECR) | No | No | Dennis et al., 2020 (Canada) |
| No | No | No | Yes (significant improvement in DASS-42 scores) | No | No | Dustan, et al., 2012 (Australia) |
| **Acceptance and Commitment Therapy (n=1)** | | | | | |  |
| Yes  (high satisfaction ratings on two forced-choice response items) | No | No | Yes  (67% of telephone  participants reduced cigarette smoking) | No | Yes  (no suicidal ideation or suicide attempts in telephone-deliered counseling) | Heffner et al., 2015 (USA) |
| **Acceptance Based Behavior Therapy for Social Anxiety Disorder (n=1)** | | | | | |  |
| Yes  (high satisfaction scores on PSS; high WAI-SF scores) | No | No | Yes  (significant reductions in psychopathology  and improvements in functioning at post-treatment and 3-month follow-up - SPAI-SP, LSAS, Brief-FNE) | No | No | Yuen et al., 2013 (USA) |
| **Anger Management Therapy (n=1)** | | | | | |  |
| Yes  (high scores on CPOSS-VA; high scores on GTAS) | No | No | Yes  (improvement in STAXI-2  anger expression and trait anger subscale scores & on NAS-T) | No | No | Morland et al., 2010 (USA) |
| **Behavioral Activation (n=1)** | | | | | |  |
| No | No | No | Yes  (similar outcomes between telemedicine and same room group: GDS, BDI, SCID) | No | No | Egede, 2015 (USA) |
| **Brief Psychosocial Behavioral Intervention (n=1)** | | | | | |  |
| No | No | No | Yes  (non-significant increase in % decrease in HRSD scores and % participants in remission in combined intervention groups) | No | No | Kirkness, et al., 2017 (USA) |
| **Positive Psychology (n=1)** | | | | | |  |
| Yes  (high acceptability rating) | No | No | Yes  (significant improvement in positive affect and optimism - LOT-R, PANAS) | No | No | Celano et al., 2020 (USA) |

Note: “Yes” indicates that the HQM dimension was measured in some way; “No” indicates that the HQM dimension was not measured.

**Abbreviations:**

ACF: Assessment of Current Functioning

ACQ: Agoraphobic Cognition Questionnaire

ADIS-4: Anxiety Disorders Interview Schedule for DSM-4

ASI: Anxiety Sensitivity Index

BAI: Beck Anxiety Inventory

BDI: Beck Depression Inventory

B-FNE: Brief Version of the Fear of Negative Evaluation Scale

B-IPF: Brief Inventory of Psychosocial Functioning

BSQ: Body Sensation Questionnaire

CALPAS: California Psychotherapy Alliance Scale

CAPS: Clinician Administered PTSD Scale

CBT: Cognitive Behavioral Therapy

CES-D: Center of Epidemiologic Studies Depression Scale

CGI: Clinical Global Impressions

CPOSS-VA: Charleston Psychiatric Outpatient Satisfaction Scale—VA PTSD Version

CPT: Cognitive Processing Therapy

CSQ-8: Client Satisfaction Questionnaire-8

CSS: Client Satisfaction Survey

CVT: Clinical Video Technology

DAS: Dyadic Adjustment Scale

DASS: Depression Anxiety and Stress Scale

DCCS: Distance Communication Comfort Scale

DISS: Sheehan Disability Scale

ECR: Experiences in Close Relationships Scale

EPDS24: Edinburgh Postnatal Depression Scale

GAD-7: General Anxiety Disorder-7

GDS: Geriatric Depression Scale

GSI: Global Severity Index

GTAS: Group Therapy Alliance Scale

HAI: Health Anxiety Inventory

HAM-A: Hamilton Anxiety Rating Scale

HAM-D: Hamilton Depression Rating Scale

HRSD: Hamilton Rating Scale for Depression

ICBT: Internet-based Cognitive Behavioral Therapy

IIRS: Illness Intrusiveness Rating Scale

LOT-R: Life Orientation Test-Revised

LSAS: Liebowitz Social Anxiety Scale

LEC: Life Events Checklist

MADRS: Montgomery-Asberg Depression Rating Scale

MCS: Mental Health Composite Score

MHI: Mental Health Inventory

MI: Mobility Inventory

MPSS: Modified PTSD Symptom Scale

NAS-T: Novaco Anger Scale-total score

OCD: Obsessive-Compulsive Disorder

OCI: Obsessive-Compulsive Inventory

P&A: Panic and Agoraphobia Scale

PANAS: Positive and Negative Affect Schedule

PASS: Perceptions About Service Scale

PCL: PTSD Checklist

PE: Prolonged Exposure

PGI: Patient Global Impression

PHQ-9: Patient/Physician Health Questionnaire-9

PI: self-report revised Padua Inventory

PSDI: Positive Symptom Distress Index

PSS: Patient Satisfaction Survey

PST: Positive Symptom Total

PTSD: Post Traumatic Stress Disorder

PSWQ: Penn State Worry Questionnaire

QLES: Quality of Life Enjoyment and Satisfaction scale

RTQ: Reaction to Treatment Questionnaire

SCID: Structured Clinical Interview for DSM-4, clinician version

SCL-20: Patient-Reported Symptom Checklist-20

SCL-90-R: Symptoms Checklist-90 Revised

SDP: Service Delivery Perceptions measure

SE-CPAQ: Self-Efficacy to Control a Panic Attack Questionnaire

SF: Medical Outcomes Study Short Form–36

SF-12: SF-12 Version 2.0 Health Survey

SPAI-SP: Social Phobia and Anxiety Inventory-Social Phobia subscale

STAI: State-Trait Anxiety Inventory

STAXI-2: State-Trait Anger Expression Inventory-2

TSAS: Telemedicine Satisfaction and Acceptance Scale

VT-Q: Videoconference Therapy Questionnaires

VOCI: Vancouver Obsessional Compulsive Inventory

WAI: Working Alliance Inventory

WAI-SF: Working Alliance Inventory-Short Form

Y-BOCS: Yale-Brown Obsessive-Compulsive Scale
